# Theoretical Approaches for Modeling the Effect of the Electrode Potential in the SERS Vibrational Wavenumbers of Pyridine Adsorbed on a Charged Silver Surface

**DOI:** 10.3389/fchem.2019.00423

**Published:** 2019-06-05

**Authors:** Daniel Aranda, Samuel Valdivia, Juan Soto, Isabel López-Tocón, Francisco J. Avila, Juan C. Otero

**Affiliations:** Andalucía Tech, Unidad Asociada CSIC, Departamento de Química Física, Facultad de Ciencias, Universidad de Málaga, Málaga, Spain

**Keywords:** SERS, Raman, pyridine, electrode potential, DFT, vibrational wavenumbers, vibrational Stark effect

## Abstract

Vibrational wavenumbers of pyridine adsorbed on a silver electrode have been correlated to the calculated ones from different theoretical approaches based on DFT methods. The vibrational tuning caused by the electrode potential has been simulated by means of pyridine-silver clusters with different densities of charge or, alternatively, under applied external electric fields. Both methodologies predict correctly a qualitative red-shift of the vibrational wavenumbers at negative potentials. As a result, harmonic frequency calculations performed at the B3LYP/LanL2DZ level of theory by using a linear [Ag_*n*_Py]^*q*^ complex model with different densities of charge (*q*_eff_ = *q/n*) have exhibited the best agreement with the experimental observations although the tuning amplitudes are overestimated. Electric fields calculations are unable to account for subtle details observed in the spectra related to the differentiated chemical nature of the metal-molecule bond at positive or negative potentials with respect to the potential of zero charge of the electrode.

## Introduction

Surface-enhanced Raman Scattering (SERS) is one of the most powerful techniques to get insight into the complex electronic structure of molecules bonded to charged metals (Weaver et al., 1985; Wolkow and Moskovits, 1992; Szekeres and Kneipp, 2019). These metal-molecule hybrid systems are involved in many scientific fields like electrochemistry or heterogeneous catalysis but they are also related to the electrical transport through single molecule junctions or are part of electronic devices for solar energy conversion. All of these scientific areas are controlled by the overall electronic structure of the metal-adsorbate complex whose chemical and physical properties can be, in many cases, tuned continuously by applying electric potentials.

The main characteristic of SERS is the huge enhancement of the Raman signal, but the spectra show very often changes in the relative intensities as well as in the vibrational wavenumbers of the bands which are modified by the electric potential in electrochemical SERS experiments. Changes in the intensities are related to the enhancement mechanism or mechanisms acting in each particular experiment (plasmonic, charge-transfer resonances, etc.) (Aroca, 2006; Kneipp et al., 2006; Le Ru and Etchegoin, 2008). But the shifts between the Raman and the SERS wavenumbers of a molecule are not dependent on these mechanisms being restricted to the ground electronic structure of the metal-molecule surface complex. Pyridine (Py) is mainly bonded to a metal atom by charge donation from the lone pair of the nitrogen to vacant orbitals of the metal. In the case of this molecule the weak chemical adsorption modifies slightly the structure of the adsorbate in such a way that the aromatic ring becomes stronger given that the donated charge has non-bonding character. As a consequence, the SERS wavenumbers of the ring vibrations of pyridine should be blue-shifted which respect to the Raman ones. This behavior allows for deducing the orientation of the adsorbate with respect to a macroscopic electrode (Soto et al., 2002) without resorting to the involvement of any SERS enhancement mechanism which is a controversial question (Moskovits, 2013; Aranda et al., 2018).

In the case of electrochemical SERS experiments, the electrode potential controls the strength of the metal-molecule bond. More positive potentials induce more positive excess of charge on the electrode, favoring charge donation from the pyridine to the metal, strengthening the ring (Avila et al., 2011a) and shifting the wavenumbers of the ring vibrations toward the blue. On the contrary, negative potentials reduces the strength of the complex and the corresponding wavenumbers should be red-shifted. This dependence of the vibrational wavenumbers observed from the electrochemical interfaces on the applied electrode potential is called usually vibrational Stark effect. For pyridine adsorbed on a silver electrode, this effect has been previously discussed by Johansson (Johansson, 2005).

Besides the effect of the electrode potential, the vibrational wavenumbers are affected by the interaction with the environment. Previous studies have shown that the wavenumber of ring breathing vibration changes with the molar fraction (Schlücker et al., 2001; Tukhvatullin et al., 2010; Kalampounias et al., 2015), demonstrating that even weak solute-solvent interactions can perturbate the electronic structure of the solute, as is observed in the experiments. When a molecule is adsorbed on electrodes these interactions change and can have a non-negligible role (Kelly et al., 2016) but the theoretical quantification of their effects in the vibrational wavenumbers remains a challenge.

**Figure 1:**
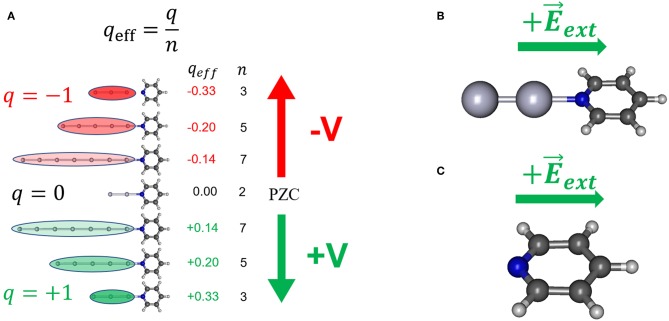
Some of the theoretical models used in simulating the effect of the electrode potential on the electronic structure of adsorbate; **(A)** the [Ag_*n*_Py]^*q*^/*q*_eff_ model; **(B)** the [Ag_*2*_Py]^*0*^/$\vec{E}$ model; **(C)** the model Py/$\vec{E}$.

This work deals with the comparative study of the performance of different theoretical methodologies for modeling the dependence of the vibrational wavenumbers of pyridine adsorbed on a charged silver interface. The experimental results obtained from SERS spectra are compared with the calculated shifts by assuming three different approaches (Figure 1): model [Ag_*n*_Py]^*q*^/*q*_eff_, a pyridine bonded to linear metal clusters with different densities of charge *q*_eff_; model [Ag_*2*_Py]^*0*^/$\vec{E}$, a pyridine bonded to a dimer neutral cluster in the presence of an external electric field and, for the sake of comparison, model Py/$\vec{E}$, the isolated molecule subjected to electric fields. Additionally, solvent effects have been considered for the different systems studied. The aim of this work is to discuss the accuracy of different theoretical approaches for predicting the vibrational wavenumber shifts of molecules bonded to metals in charged interfaces in order to know the effect of the adsorption and the electric potentials in the overall electronic structure of metal-molecule hybrids.

## Materials and Methods

### The SERS Measurements

Electrochemical SERS spectra have been recorded using a three electrodes cell controlled by a CH model 600E potentiostat, with a platinum counter electrode, an Ag/AgCl/KCl (sat.) reference electrode to which all the electrode potentials are referred to, and a pure silver working electrode. This latter was polished with 0.30 and 0.05 μm alumina (Buëhler) and electrochemically activated by using a 0.1 M KCl aqueous solution as electrolyte by maintaining the electrode potential at −0.5 V, and then subjecting it to seven 2 s pulses at +0.6 V in order to produce the necessary surface roughness for SERS enhancement.

Pyridine was purified by distillation in vacuum and a 0.1/0.1 M aqueous solution of pyridine/KCl was used to record the SERS spectra. The water of the solutions was of Milli-Q quality (resistivity over 18 MΩcm).

Raman and SERS spectra have been recorded with 1 cm^−1^ resolution by using a Renishaw Invia micro-Raman spectrometer and the excitation line of 514.5 nm from an Ar^+^ gas laser. The microscope has an adapted objective (f: 30 mm) to work in macro conditions. To avoid overheating during the measurement of Raman spectra neutral density filters with an optical throughput of 0.5 and 1% were used and the laser power at the sample position was 0.1 and 5 mW, respectively.

### DFT Calculations

Electronic structure calculations were performed using Density Functional Theory (DFT) as implemented in the Gaussian09 package (Frisch et al., 2010). Three different functionals B3LYP (Becke, 1993; Stephens et al., 1994), PW91 (Perdew et al., 1996) and M06-HF (Zhao and Truhlar, 2006) have been checked in combination with several basis sets. The LanL2DZ (Hay and Wadt, 1985a,b,c) effective core potential was selected for silver atoms, whereas for pyridine the same LanL2DZ pseudopotential (DV95V, Dunning and Hay, 1977), 6-31G(d) (Ditchfield et al., 1971), and 6-311G(d,p) (Krishnan et al., 1980; McLean and Chandler, 1980) basis sets have been also tested.

B3LYP and PW91 functionals usually provide accurate vibrational wavenumbers, whose shifts caused by the adsorption is the main focus of this work. The long-range corrected M06-HF functional is less reliable in computing vibrational wavenumbers as it remarkably overestimates them. Nevertheless, this method should be expected to return more reliable energy values for some particular cases than the non-corrected functionals like B3LYP (Avila et al., 2011a), and we also used it in this study considering that it was previously used to quantify the presence of metal-to-molecule charge transfer resonances in similar systems.

Solvent effects were taken into account using the PCM (Polarizable Continuum Model, Tomasi et al., 2005) model as implemented in Gaussian 09 with standard parameters. The main drawback of implicit models is that they cannot reproduce properly specific interactions like hydrogen bonding, but they are expected to be small for chemically adsorbed pyridine since the nitrogen atom is not available for hydrogen bonding.

The effect of the electrode potential has been modeled in the calculations by assuming two different approaches briefly described below: charged metal clusters and external electric fields. The electrode potential tunes the surface excess of charge of the electrode. Consequently, it modifies also the ionic structure of the electrolyte part of the double layer which can be simulated by means of an electric field. Both factors, metal charges and external electric fields, modify the electronic structure of the metal-molecule hybrid and therefore, shift the vibrational wavenumbers of the adsorbate.

### The Charged Metal Cluster Models

In order to model the effect of the metal excess of charge on the metal-molecule surface complex [Ag_*n*_Py]^*q*^ systems formed by linear metal clusters with different sizes (*n* = 2, 3, 5, 7) and charges (*q* = 0 for *n* = 2, and *q* = ± 1 a.u. for odd *n*) (Figure 1A) have been used. These complexes allow for defining the effective density of charge of the cluster as *q*_eff_ = *q*/*n* (atomic units) which ranges from +0.33 to −0.33 a.u. in this series. Previous studies indicate that this range of *q*_eff_ can be correlated with electrochemical SERS results recorded from 0.0 to −1.5 V (Román-Pérez et al., 2014a; Roman-Perez et al., 2015).

*q*_eff_ is the average density of charge of the cluster and is the microscopic analogous of the macroscopic surface excess of charge of a metal, *q*′ (C/cm^2^), which is more or less linearly tuned by the electrode potential. In this way, the neutral Ag_*2*_ cluster with *q* = 0 would simulate the case of pyridine adsorbed on a neutral metal surface, i.e., at the potential of zero charge of the electrode (**V**_PZC._) Therefore, positively charged clusters ([Ag_*n*_Py]^+1^, *q*_eff_ > 0) should correspond to electrode potentials more positive than **V**_PZC_ (+**V**) whereas negative clusters ([Ag_*n*_Py]^−1^, *q*_eff_ < 0) simulate potentials more negative that **V**_PZC_ (–**V**). For a polycrystalline silver electrode **V**_PZC_ lies in the range of −0.8 to −0.9 V (Hupp et al., 1983; Chen and Otto, 2005). For convenience, the potential of −0.7 V has been considered as the **V**_PZC_ of the rough silver electrode given that is the center of the experimental range of potentials scanned in this work.

A rough metal surface shows a lot of different local structures at an atomic scale where a single molecule can be bonded. This gives an unaffordable range of possibilities for the geometries of the surface complex, each of them having different properties and specific interactions. To avoid selecting arbitrarily one of them we have considered the simplest case where pyridine is bonded to a terminal atom of the linear cluster through the nitrogen atom. In spite of its simplicity, this model has proved its usefulness in analyzing the complex behavior in the SERS spectra of benzene-like molecules, which have been considered in our previous works (Arenas et al., 2002; Avila et al., 2011a,b and references therein).

### The External Electric Field Models

An alternative and widely used strategy consist of simulating the effect of the electrode potential by means of external electric fields ${\vec{E}}_{ext}$ applied, in this case, to isolated pyridine (model Py/$\vec{E}$, Figure 1C) or to the neutral [Ag_*2*_Py]^*0*^ complex (model [Ag_*2*_Py]^*0*^/$\vec{E}$, Figure 1B) by using the FIELD keyword in Gaussian 09 calculations. Fields has been imposed by assuming a single component perpendicular to the surface along the symmetry axis of the molecule. It is important to clearly define the sign of ${\vec{E}}_{ext}$ by considering the criterium of the particular computer program used (Aranda et al., 2017). The sign of ${\vec{E}}_{ext}\;$has been selected in such a way that positive fields ($+\vec{E}$) polarize the negative electronic cloud of the adsorbate toward the electrode and, therefore, it corresponds to positive potentials or positive excess of charge on the metal (+**V**). Calculated wavenumbers of pyridine at zero-field are those of the isolated molecule, but they must be correlated to the experimental ones recorded in the SERS at **V**_PZC_ which are perturbed by the chemical adsorption on a neutral metal surface simulated through the neutral [Ag_*2*_Py]^*0*^ complex. However, a comparison between the results of the simple linear model [Ag_*2*_Py]^*0*^/$\vec{E}$ and model [Ag_*20*_Py]^*0*^/$\vec{E}$, where pyridine is bonded to tetrahedral ${\text{Ag}}_{\text{20}}^{\text{0}}$ cluster (Zhao et al., 2005; Zhao and Chen, 2013), will be presented in order to discuss the effect of the cluster size and geometry.

The selection of the range of applied ${\vec{E}}_{ext}$ to reproduce the electrochemical SERS experiments recorded from 0 to −1.4 V is not trivial. This approach has been very frequently used in the earlier similar studies. For instance, Mohammadpour et al. (2017) used an asymmetric range of 120·10^−4^ a.u. by using a planar-triangular ${\text{Ag}}_{\text{6}}^{\text{0}}$ cluster to reproduce the trends observed on the SERS intensities of pyridine recorded by us on silver in the range −0.5 to −1.2 V. Zhao and Chen (2013) studied pyridine on a gold electrode modeled as a Au_*20*_ tetrahedral cluster using a range of ±10·10^−4^ a.u. in order to reproduce the SERS intensities focused in the 1,000 cm^−1^ region. Differently from the others, in this study we screened a larger range of the applied external electricity field $\Delta {\vec{E}}_{ext}=$ ±120·10^−4^ a.u. to have an overall view on its effect on the fundamental bands observed in the spectra of pyridine.

### Correlating Parameters of Theoretical Models With the Electrode Potential

The calculation results have indicated that, at the B3LYP/LanL2DZ level of theory, the effect of the electric field applied in the range of ±120·10^−4^ a.u. on the calculated properties of silver-pyridine complex models is comparable to those calculated of the charged silver-pyridine models with Δ*q*_eff_ ± 0.33 a.u. For instance, the injected charge from pyridine to the metal in the extreme negative case amounts to −0.072 and −0.071 a.u. in the models [Ag_*2*_Py]^*0*^/$\vec{E}$, with ${\vec{E}}_{ext}\;$ = −120·10^−4^ a.u., and [Ag_*n*_Py]^*q*^/*q*_eff_, with *q*_eff_ = −0.33 a.u., respectively. In turn, values of −0.268 and −0.273 a.u. are obtained at the positive side with ${\vec{E}}_{ext}\;$ = +120 10^−4^ a.u. and *q*_eff_ = +0.33 a.u., respectively. The difference between the stability of the metal-molecule surface complex between the most negative and the most favorable positive potentials can be estimated by comparing the relative energies of the systems of both models at the extreme values of the respective ranges, giving Δ*E*_±_ of 46.33 and 45.75 Kcal/mol for models [Ag_*2*_Py]^*0*^/$\vec{E}$ and [Ag_*n*_Py]^*q*^/*q*_eff_, respectively. Furthermore, the only available explanation so far of the huge energy gain (G = Δ*E*_*CT*_ / ΔV up to 3–5 eV/V, Otto et al., 1984; Cui et al., 2010; Aranda et al., 2017) observed in SERS when the electrode potential (**V**) tunes the energies of the metal-to-molecule charge transfer states (E_CT_) requires that the usual range of experimental electrode potentials Δ**V** = **V**_**+**_–**V**_**−**_ = 1.5 V is again correlated to the calculated E_CT_ energies of [Ag_*n*_Py]^*q*^/*q*_eff_ complexes with *q*_eff_ = +0.33 and −0.33 a.u. From the two [Ag_*3*_Py]^±1^ systems a value of Δ*E*_CT_ = 3.63 eV is obtained, while the model [Ag_*2*_Py]^*0*^/$\vec{E}$ provides an estimation of only 1.99 eV from $\Delta {\vec{E}}_{ext}$ = ±120·10^−4^ a.u. This is a lack of the electric field approach given that ΔE_CT_ is too small to account for the experimental energy gain (Aranda et al., 2017).

**Figure 2:**
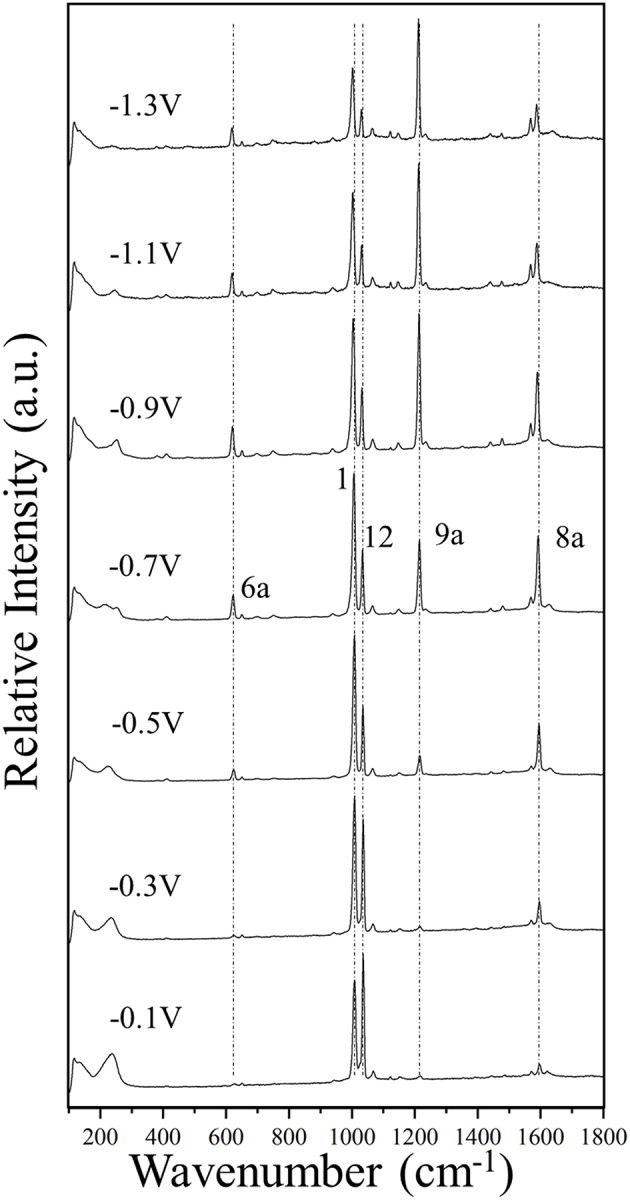
The SERS spectra of pyridine (0.1 M) adsorbed on silver, recorded at different electrode potentials by using a laser source of 514.5 nm wavelength.

Finally, the strong selective enhancement of mode 9a in the SERS of pyridine recorded at very negative electrode potentials (see Figure 2 and Supplementary Figure S1) can be only explained on the basis of a Raman resonance process up to intracluster electronic excitations in the case of the most negatively charged [Ag_*n*_Py]^−1^ complexes (Roman-Perez et al., 2015). This means that the experimental conditions at −1.2 or −1.4 V can be only reproduced by theoretical models with *q*_eff_ = −0.33 a.u.

The previous discussion in which the experimental and theoretical parameters are related to one other allows for correlating the respective ranges of the experimental and theoretical parameters, such as electrode potential (Δ**V** = 1.5), charge (Δ*q*_eff_ = ±0.33 a.u.) and electric fields ($\Delta {\vec{E}}_{ext}$ = ±120·10^−4^ a.u.).

### Wavenumber Shifts Arising From the Molecular Adsorption

To study the changes of the electronic structure of the adsorbate, the wavenumber shift Δυ_*i*_(*V*) of each vibrational mode *i* at a particular potential V has been computed as follows:

$$\begin{array}{l} \Delta \upsilon_{i}\left(V\right)=\upsilon_{i}\left(V\right)-\;\upsilon_{i}\left(Ref\right) \end{array}$$

Where υ_*i*_(*V*) is the absolute wavenumber of the mode *i* at the potential V and υ_*i*_(*Ref*) is the wavenumber of the mode *i* in the selected reference conditions. This work focuses on the adsorption phenomenon, so the experimental data of pure liquid pyridine have been selected as a reference. Therefore, Δυ_*i*_(*V*) contains adsorption and solvation effects, the strength of the adsorption being modulated by the electrode potential. In the case of theoretical results, **V** is simulated by *q*_eff_ or ${\vec{E}}_{ext}$ and the reference was the calculated wavenumbers for a single pyridine molecule in a pyridine PCM environment, while water parameters has been used for solvent correction in the PCM calculations of silver-pyridine complexes.

## Results and Discussion

The effect of the adsorption on a neutral surface will be discussed firstly by comparing the wavenumbers of liquid pyridine with those of the SERS at −0.7 V and these results will be compared with the theoretical calculations carried out with and without solvent corrections. Thereafter, the effect of the electrode potential on the wavenumber shifts will be discussed by comparing the experimental data with three sets of calculations carried out under different approaches: effect of the charge of the metal clusters (model [Ag_*n*_Py]^*q*^/*q*_eff_), effect of electric fields in a complex where a molecule is adsorbed on the ${\text{Ag}}_{\text{2}}^{\text{0}}$ neutral cluster (model [Ag_*2*_Py]^*0*^/$\vec{E}$) and effect of electric fields in the wavenumbers of an isolated pyridine (model Py/$\vec{E}$).

### The Experimental SERS Spectra of Pyridine

Figure 2 and Supplementary Figure S1 show the potential-dependent SERS of pyridine adsorbed on silver electrode, on steps of 0.2 V and 0.1 V, respectively. Some vertical dotted lines have been added to the main SERS bands corresponding to the totally symmetric modes 6a, 1, 12, 9a, and 8a recorded at around 622, 1,006, 1,034, 1,215, and 1,592 cm^−1^, respectively. The wavenumbers of the strongest SERS bands measured in the complete set of spectra recorded in steps of 0.1 V are reported in the Supplementary Table S1. The experimental Δυ_*i*_(*V*) selected by Johansson (2005) of only an electrochemical SERS spectrum for pyridine are very similar to our results obtained at the most positive potentials of 0 or −0.1 V.

The general trend is that for potentials more positive than −0.5 V the respective wavenumbers remains almost constant, while they are quasi-linearly red-shifted as the potential is made more negative. Some exceptions are the vibrational modes 18a and 6b of 1,067 and 650 cm^−1^, respectively, which are almost insensitive to the potential.

### The Changes Occurred at the Vibrational Modes of Pyridine Depending on the Molecular Adsorption on a Neutral Surface

**Figure 3:**
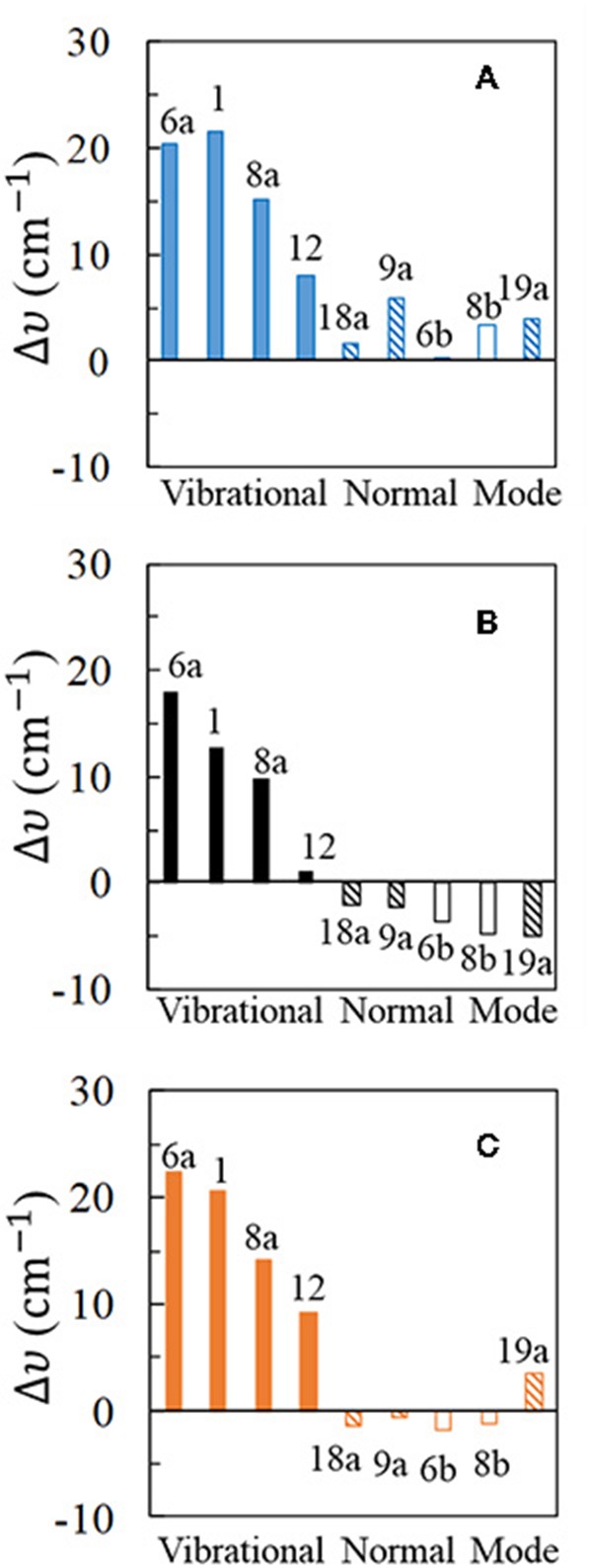
Wavenumber shifts (Δυ) determined for the most representative normal modes of pyridine: **(A,C)** show the shifts calculated at B3LYP/LanL2DZ level of theory for the [Ag_*2*_Py]^*0*^ complex model in vacuum and for the [Ag_*2*_Py]^*0*^ complex model in water environment where the solvent effects are taken into account by using the PCM (solvent = water), respectively; **(B)** shows the experimental shifts when an electrode potential of **V**_PZC_ = −0.7 V is used in the SERS measurement.

As previously pointed, the experimental conditions corresponding to the potential of charge zero has been assigned to the SERS recorded at **V**_PZC_ = −0.7 V. Figure 3 compares experimental Δυ_*i*_ obtained from the pure liquid Raman and the SERS recorded at this potential with the calculated Δυ_*i*_ from isolated pyridine and for the [Ag_*2*_Py]^*0*^ complex at the B3LYP/LanL2DZ level of theory, both with and without considering solvent effects with PCM. As can be seen, the harmonic frequency calculations produce results well agreeing with the observed general trend despite they are carried out by using a rather simple complex model [Ag_*2*_Py]^*0*^.

The experimental results (Figure 3B) show large and positive shifts for the strong SERS modes 6a, 1, and 8a (+18, +13, and +10 cm^−1^, respectively), and a small blue-shift for mode 12 (+1.2 cm^−1^). All other vibrations show moderate or small red-shifts, showing mode 19a the largest displacement (−5 cm^−1^). Pyridine vibrations can be classified in different types according to the nature and the amount of the wavenumber shifts: type I for A_1_ vibrational modes mainly localized on the ring (6a, 1, 8a, and 12), type II for A_1_ modes involving hydrogen motion or ring deformations (19a, 9a, and 18a) and finally type III for the B_2_ symmetry modes 8b and 6b. Animations of each fundamental can be found on Supplementary Material.

Harmonic frequency calculations of the [Ag_*2*_Py]^*0*^ complex predict a blue-shift for all the vibrations (Figure 3A), the most sensitive ones to the adsorption being type I in agreement with the experimental results. However, Δυ_*i*_ for mode 12 is overestimated and the negative shifts of type II and III are not well-reproduced. PCM results improve the agreement with the experiment. The large blue-shifts for type I, are preserved and remain overestimated. Types II and III now show negative displacements, except for vibration 19a. Generally speaking, PCM methodology reduces the large shifts predicted by the DFT calculations as previously detected (Brewer and Aikens, 2010). From the results of the five strongest SERS fundamentals (type I plus mode 9a) an overestimation of 50% is obtained for the calculated shifts: Δυ_*calc*_ = 1.5 Δυ_exp_.

Very similar behavior is observed with the PW91 functional, while M06-HF predictions are somewhat worse (Supplementary Figure S2). Generally speaking, B3LYP seems to be the most reliable one amongst the three compared functionals. It should be noted that the experimental shifts in Figure 3B are dependent on the rather arbitrary potential selected as **V**_PZC_ and the agreement between calculated and experimental results could be improved if a more negative SERS spectra than −0.7 V would have been chosen as reference.

For completeness, three different basis sets for pyridine have been checked in combination with the three functionals: D95V (LanL2DZ), 6-31G(d), and 6-311G(d,p), respectively, and the corresponding results can be compared by examining Figure 3 and Supplementary Figures S2–S4. As can be seen, 6-31G(d) and 6-311G(d,p) results are very similar, improving both the predictions obtained with the smaller LanL2DZ basis set. All the calculated shifts for the most characteristic type I modes are reduced, in agreement with the experimental results, as well as the relative amount of Δυ_*i*_ for vibrations 6a and 1. Finally, B3LYP and PW91 shifts calculated with the 6-31G(d) basis set for pyridine are negative for the weaker type II and III set of bands in the PCM calculations. However, the red-shift of −5.0 cm^−1^ observed for mode 19a still remains not well-reproduced.

### The Changes Occurred at the Vibrational Modes of Pyridine Depending on the Electrode Potential

The tuning of the wavenumbers by the electrode potential is discussed in this section being mainly restricted to the behavior of the five strongest SERS bands which corresponds to type I vibrations plus mode 9a.

Once the molecule is adsorbed, the vibrational wavenumbers are tuned by the electrode potential in such a way that positive potentials favors charge donation from pyridine to the metal and, therefore, the surface complex becomes stronger and the bands are blue-shifted. This tuning of the experimental wavenumbers Δυ_*V*_ originated by the applied potential is referred to the absolute SERS wavenumbers recorded in the reference spectrum at −0.7 V as:

$$\begin{array}{l} \Delta \upsilon_{V}=\upsilon_{V}-\upsilon_{PZC\;=\;-0.7\;V} \end{array}$$

**Figure 4:**
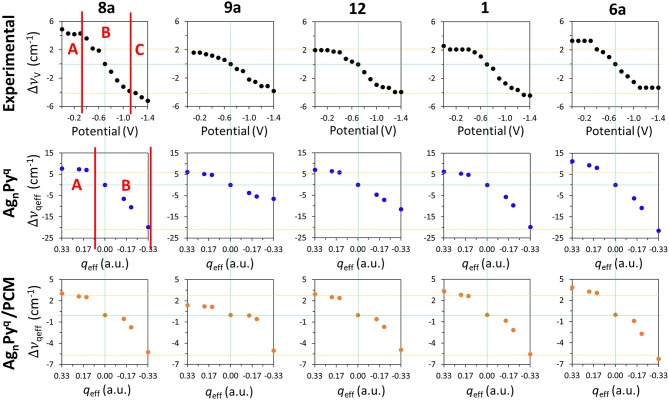
Comparison of the wavenumbers shifts observed in the SERS spectra of pyridine which are tuned by the electrode potential with those calculated at the B3LYP/LanL2DZ level of theory for the [Ag_*n*_Py]^*q*^ complex models with different *q*_eff_ in vacuum and in solvent environments by using the PCM (solvent = water).

Figure 4 shows the Δυ_*V*_ values for the main set of vibrational modes (type I plus mode 9a). As can be seen, the amplitude of the tuning for the most shifted ones (modes 8a and 6a) among them ranges from +6 to +10 cm^−1^. All of them show the expected behavior and are red-shifted as the electrode potential is more negative. It has to be stressed that a SERS spectrum recorded at a particular potential can contain contributions from an ensemble of molecules bonded to a distribution of local sites characterized by different densities of charge. Therefore, the height and width of a SERS band contain heterogeneous contributions from different molecules bonded to an unknown distribution of atomic sites of the rough electrode surface, each one of them being characterized by a particular excess of charge. This causes smoothing of the experimental dependence of the wavenumbers on the potential. The wavenumbers are more or less insensitive to the potential at the most positive values (0.0 to −0.4 V) showing a very small or near zero slope (region A in Figure 4). This is a rather surprising result given that the silver-pyridine complex should be strengthened as the density of charge of the metal increases and, therefore, the corresponding wavenumbers should be continuously blue-shifted. This behavior can be also seen in the case of pyrazine in spite of the smaller number of potentials scanned (Soto et al., 2002). Region B show the expected behavior with a rather linear positive slope, while the most negative potentials also show again a reduced slope (region C). This behavior of region C is probably due to the desorption of pyridine at very negative potentials. The relative intensities of the SERS bands observed at the potentials of −1.1, −1.2, and −1.3 V remain almost constant (Figure 2 and Supplementary Figure S1), indicating that the limit of the most negative potential which can enable to keep the metal-molecule binding has been surpassed. Potentials more negative than such limit produce the dissociation of the surface complex and, therefore, the spectra of region C could be produced by the same kind of very weak complexes whose number is being diminished as the electrode potential is even more negative. The absolute intensities of the SERS become weaker at these potentials, but the relative intensities of the bands are not affected once such negative limit is reached.

### The Results Obtained From the [Ag*_*n*_*Py]*^*q*^* Cluster Model

The shifts observed at the experimental vibrational wavenumbers of pyridine Δυ_*V*_ which are tuned by the applied potential, and those calculated for the [Ag_*n*_Py]^*q*^ model complexes at the B3LYP/LanL2DZ level of theory by taking [Ag_*2*_Py]^*0*^ as a reference, are shown in Figure 4.

$$\begin{array}{l} \Delta \upsilon_{qeff}=\upsilon_{{\left[Ag_{n}Py\right]}^{q}}-\;\upsilon_{{\left[Ag_{2}Py\right]}^{0}} \end{array}$$

As can be observed, the general behavior of the dependence of the wavenumbers on *q*_eff_ agrees qualitatively very well with the experimental results. While the largest shifts are obtained for the vibrational modes 8a and 6a in the calculations, the shifts predicted for the modes 9a, 12, and 1 are smaller as observed in the experimental measurements. The relative slopes obtained from the wavenumbers calculated for these five vibrational modes exhibit a good agreement with those obtained from the observed wavenumbers. All Δυ_*qeff*_ are more or less constant for positive densities of charge (*q*_eff_ > 0), so reproducing the characteristics of the experimental region A. This could be related to the previously described bi-modal behavior of the electronic structure of complexes formed by organic molecules (A) bonded to charged metal clusters (M^q^) (Román-Pérez et al., 2014a). In the case of charged adsorbates like the isonicotinate anion this effect is very striking, and the structure of complexes formed with positive clusters are almost insensitive to *q*_eff_, while the properties of complexes in neutral (*q*_eff_ = 0) or negative (*q*_eff_ < 0) cases are very dependent on the electrode density of charge. In the first case, a strong (attractive) chemisorbed hybrid species is formed (A^−^-M^+^) and its electronic structure is almost insensitive to the electrode potential. In the second case (A^−^-M^0^ and A^−^-M^−^) the physisorbed (repulsive) complex is weak and its properties can be smoothly tuned by the charge of the metal. Pyridine is a neutral molecule and can be considered as a case of physisorption in all the potential range (Avila et al., 2011a). Therefore, this duality should be less evident in pyridine than in cases of charged adsorbates, but the experimental and calculated results point to a minor but significant change in the chemical nature of silver-pyridine hybrids at positive or negative/neutral charges of the metallic surface.

The [Ag_*3*_Py]^*-1*^ is the most negative case where the complex is almost dissociated as previously reported with M06-HF calculations (Avila et al., 2011a) and marks the negative limit of region B in Figure 2. This explains why the B3LYP/LanL2DZ calculated wavenumbers at the corresponding value of *q*_eff_ = −0.33 a.u. are very similar to those of the isolated pyridine.

Although a qualitative agreement between Δυ_*V*_ and Δυ_*qeff*_ is found in Figure 4, the calculated amplitudes of the effect of the excess of charge of the metal on the wavenumbers of this set of five vibrations are strongly overestimated by B3LYP/LanL2DZ calculations (−28, −13, −19, −26, and −33 cm^−1^) with respect to the SERS results (−10, −5 −6, −6, and −7 cm^−1^): Δυ_*qeff*_ = 3.5 Δυ_*V*_. PCM calculations reduce significantly this discrepancy. This may be because the metal cluster is also inside the cavity and the counter charges located around the metal in the PCM calculations diminish the effect of *q*_eff_ of the respective clusters. The amplitudes calculated by considering the effect of the solvent are only overestimated by about 20%: Δυ_*qeff*_ = 1.2 Δυ_*V*_. Unfortunately, this numerical improvement is not accomplished by appropriate dependences on *q*_eff_. PCM is unable to account for the differentiated behavior of the experimental shapes of this set of five fundamentals (Figure 4). Now all vibrations show almost the same shape and amplitude, and only the positive part of the curve of mode 9a differs slightly from the remaining ones. Moreover, a discontinuity can be appreciated in the results for the neutral complex [Ag_*2*_Py]^*0*^ indicating that the PCM method does not modify the properties of charged species in a similar way than the neutral ones.

Concerning the dependence of the results on the level of theory, the three functionals predict similar behaviors, although a poorer performance can be seen in the case of M06-HF. With respect to the basis set size, the very large Δυ_*qeff*_ values provided for this model [Ag_*n*_Py]^*q*^/*q*_eff_ with the LanL2DZ basis are slightly reduced by using the 6-31G(d) or 6-311G(d,p) basis for the pyridinic atoms. As an example, Supplementary Figure S5 compares the experimental Δυ_*V*_ and the B3LYP calculated Δυ_*qeff*_ using three different basis sets, LanL2DZ, def2-TZVPP (Weigend and Ahlrichs, 2005) and LanL2DZ/6-31G(d), respectively. As can be seen, B3LYP/LanL2DZ and def2-TZVPP calculations look like very similar and all of them overestimate the amount of the tuning (slopes and amplitudes) of the electrode potential. The [Ag_*n*_Py]^*q*^ complexes become even more repulsive at the B3LYP/LanL2DZ/6-31G(d) level and dissociates in the case of *q*_eff_ = −0.33 a.u. giving a very large N-Ag distance at the end of the geometry optimization process. This is the cause of the anomalous values shown by modes 9a and 12 while vibrations 8a, 1, and 6a are well-behaved.

### The Results Obtained From the Py/$\overset{\to }{\text{E}}$ and [Ag_*2*_Py]${}^{0}/\overset{\to }{\text{E}}$ Models

**Figure 5:**
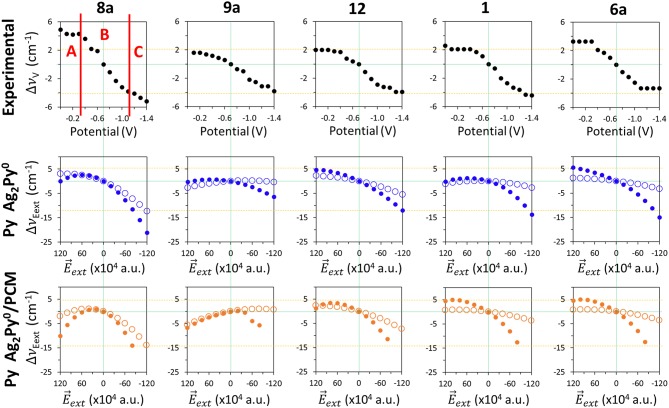
Comparison of the wavenumbers shifts observed in the SERS spectra of pyridine which are tuned by the electrode potential with those calculated at the B3LYP/LanL2DZ level of theory by applying external electric fields for the isolated pyridine (empty circles) and for the [Ag_*2*_Py]^*0*^ complex model (full circles) in vacuum and in solvent environments by using the PCM (solvent = water).

External electric fields polarize the electronic cloud of a molecule along the direction of the field with respect to the equilibrium structure. Figure 5 show the effect of ${\vec{E}}_{ext}$ in the calculated B3LYP/D95V (LanL2DZ) wavenumber shifts $\Delta \upsilon_{{\vec{E}}_{ext}}$for isolated pyridine (empty circles):

$$\begin{array}{l} \Delta \upsilon_{{\vec{E}}_{ext}}=\upsilon_{Py,\;{\vec{E}}_{ext}}-\upsilon_{Py,\;{\vec{E}}_{ext}\;=\;0} \end{array}$$

As can be seen, all the five discussed modes are smoothly shifted in the ±120·10^−4^ a.u. range of fields with a curved shape resembling parabolic sections. These curves are not symmetric with respect to ${\vec{E}}_{ext}=0$ a.u. because pyridine is not centrosymmetric like benzene or pyrazine, being this behavior much more evident in the case of mode 8a, which shows the largest amplitude amounting to −15 cm^−1^. The tuning of the wavenumbers by the field in the case of the remaining modes 1, 12, 6a, and 9a is smaller (around −5 cm^−1^), but the trend is just the opposite to the expected one in the case of vibration 9a. In the same Figure 5 (full circles) the calculated values for the [Ag_*2*_Py]^*0*^ complex can be also compared:

$$\begin{array}{l} \Delta \upsilon_{{\vec{E}}_{ext}}=\upsilon_{{\left[Ag_{2}Py\right]}^{0},\;{\vec{E}}_{ext}}-\upsilon_{{\left[Ag_{2}Py\right]}^{0},\;{\vec{E}}_{ext}=\;0} \end{array}$$

The presence of the ${\text{Ag}}_{\text{2}}^{\text{0}}$ cluster makes the electronic density of pyridine much more polarizable resulting in a larger sensitivity of the wavenumbers to the field. The amplitudes are increased up to −21, −6, −17, −14, and −21 cm^−1^ for the respective 8a, 9a, 12, 1, and 6a modes, and the parabolic trend is more evident, especially in the cases of vibrations 8a, 9a, and 1 which show a maximum at +60 10^−4^ a.u. Once again, the amplitude of the predicted tuning is overestimated with respect to the experimental results and the behavior is similar regardless the functional or the basis set used.

Any improvement can be seen in the PCM results also shown in Figure 5. The curvature is now more evident, notably in the case of mode 8a, vibration 9a shows two differentiated and unexpected change of trend before and after of +20 10^−4^ a.u. and the [Ag_*2*_Py]^*0*^ complex dissociates at fields more negative than −80 10^−4^ a.u. Therefore, electric field calculations do not reproduce well the observed results given that neither the experimental region A, with near zero slope, nor region B, with a linear dependence, can be distinguished. Moreover, the curved profiles do not allow for quantifying the dependence on the field by means of a single numerical slope.

A similar assessment can be applied to the results obtained for the effect of electric fields in the case of pyridine bonded to a larger tetrahedral ${\text{Ag}}_{\text{20}}^{\text{0}}$ complex. Two different geometries have been studied with the molecule bonded to a vertex (V-complex) or to the center of a face (S-complex) (Supplementary Figure S6). B3LYP/LanL2DZ results for these systems can be compared to the experimental SERS results and the calculated shifts for model Ag_*2*_Py^*0*^/$\vec{E}$ in Supplementary Figure S6. Full optimization of the geometries of both Ag_*20*_Py^*0*^ hybrids have the problem that pyridine losses the original orientation at negative fields when the metal-molecule bond is too weak and breaks. In this circumstance, the dipole of pyridine is realigned along the strong negative field, migrates to another site or dissociates, depending on the particular complex and the field strength. Anyway, the general behavior shown by the two large complexes [Ag_*20*_Py]^*0*^ and the simpler [Ag_*2*_Py]^*0*^ system looks like very similar.

Concerning the remaining A_1_ (19a and 18a) and B_2_ (8b and 6b) fundamentals all of them are very weak in SERS and the respective wavenumbers show a little dependence on the electrode potential, in rough agreement with the corresponding B3LYP/LanL2DZ results (Supplementary Figure S6). It should be stressed that both B_2_ bands do not show the blue-shift theoretically predicted from the wavenumbers calculated at negative electrode potentials. This behavior of pyridine differs from the one reported for the SERS spectra of pyrazine (Soto et al., 2002), at which both the observed and calculated wavenumbers indicate a blue-shift. The interested reader can reach a complete set of the figures (Supplementary Figures S8–S28) presented in this study in the **Supplementary** section where the observed wavenumber shifts Δυ_*i*_(*V*) are compared with those calculated at the different levels of theory.

## Concluding Remarks

This work discusses the dependence on the applied electrode potential of the vibrational wavenumbers of pyridine adsorbed on a metal surface in the light of calculation results obtained at different levels of theory. The experimental results have indicated small but significant shifts for the wavenumbers of some respective bands observed in the SERS spectra of a pyridine-silver interface. The effect of the electrode potential has been considered in the DFT calculations by means of pyridine-silver [Ag_*n*_Py]^*q*^ complexes with different densities of charge (*q*_eff_) or by applying external electric fields to a neutral [Ag_*2*_Py]^*0*^ complex. The dependence of the results with the functional (B3LYP, PW91 and M06-HF) the basis set size (LanL2DZ and combined LanL2DZ/6-31G(d) or LanL2DZ/6-311G(d,p) sets for silver/pyridine atoms, respectively) has been discussed. B3LYP and PW91 provide similar results while M06-HF shows a poorer performance. Larger basis set does not improve the quality of the predictions as well as the size of the metal cluster in models using electric fields, given that [Ag_*20*_Py]^*0*^ complexes add nothing to predictions derived from the simple [Ag_*2*_Py]^*0*^ hybrid. As a conclusion, it does not seem worth the use of more realistic models of the metal surface in the theoretical estimations of the here discussed properties.

Although these conclusions, based on the linear [Ag_*n*_Py]^*q*^ complex models which are applied at the B3LYP/LanL2DZ level of theory, need to be confirmed also for the other similar molecular systems, they have shown that the simple model used here satisfactorily reproduce the shifts observed at the vibrational wavenumbers of the pyridine-silver complexes. Moreover, this model is also able to account for the small or near zero slope of the experimental tuning of the wavenumbers at positive electrode potentials (region A) as well as for the linear red-shift observed at more negative potentials than **V**_PZC_ (region B). However, theoretical predictions are overestimated and need to be numerically corrected. Summarizing, DFT calculations on the properties of linear [Ag_*n*_Py]^*q*^ complexes have proved once again its usefulness for understanding complex SERS results like the enhancement of selective in-plane enhancement fundamentals of aromatic molecules under charge transfer (Avila et al., 2011b) or plasmon-like (Roman-Perez et al., 2015) resonant processes, the effect of the symmetry of the surface complex in the SERS selection rules (Centeno et al., 2006, 2012), the dual electronic structure of charged molecules bonded to charged metals (Román-Pérez et al., 2014b), the activity of the out-of-plane bands in SERS (Aranda et al., 2018), the huge energy gain of the electrode potential in tuning the energies of the charge transfer states (Román-Pérez et al., 2014a) or the wavenumber shifts of the vibrations under adsorption on neutral or charged metal surfaces.

## Data Availability

The raw data supporting the conclusions of this manuscript will be made available by the authors, without undue reservation, to any qualified researcher.

## Author Contributions

DA, SV, JS, and FA carried out DFT calculations. IL-T recorded the SERS spectra. JO and FA coordinated and designed the research. All authors contributed to write the sections of the manuscript and to revise it, read and approved the submitted version.

### Conflict of Interest Statement

The authors declare that the research was conducted in the absence of any commercial or financial relationships that could be construed as a potential conflict of interest.
