# Supplementary material for: Theoretical Approaches for Modeling the Effect of the Electrode Potential in the SERS Vibrational Wavenumbers of Pyridine Adsorbed on a Charged Silver Surface
Source: Front Chem. 2019 Jun 5;7:423. doi: 10.3389/fchem.2019.00423 (PMC6560080; doi:10.3389/fchem.2019.00423)
Supplement: Supplementary file 1 [file Data_Sheet_1.pdf]

Supplementary Material

Theoretical Approaches for Modeling the Effect of the Electrode Potential in the SERS Vibrational Wavenumbers of Pyridine Adsorbed on a Charged Silver Surface

Daniel Aranda, Samuel Valdivia, Juan Soto, Isabel López-Tocón, Francisco J. Avila,* Juan C. Otero*

*** Correspondence:** Juan Carlos Otero.: jc_otero@uma.es

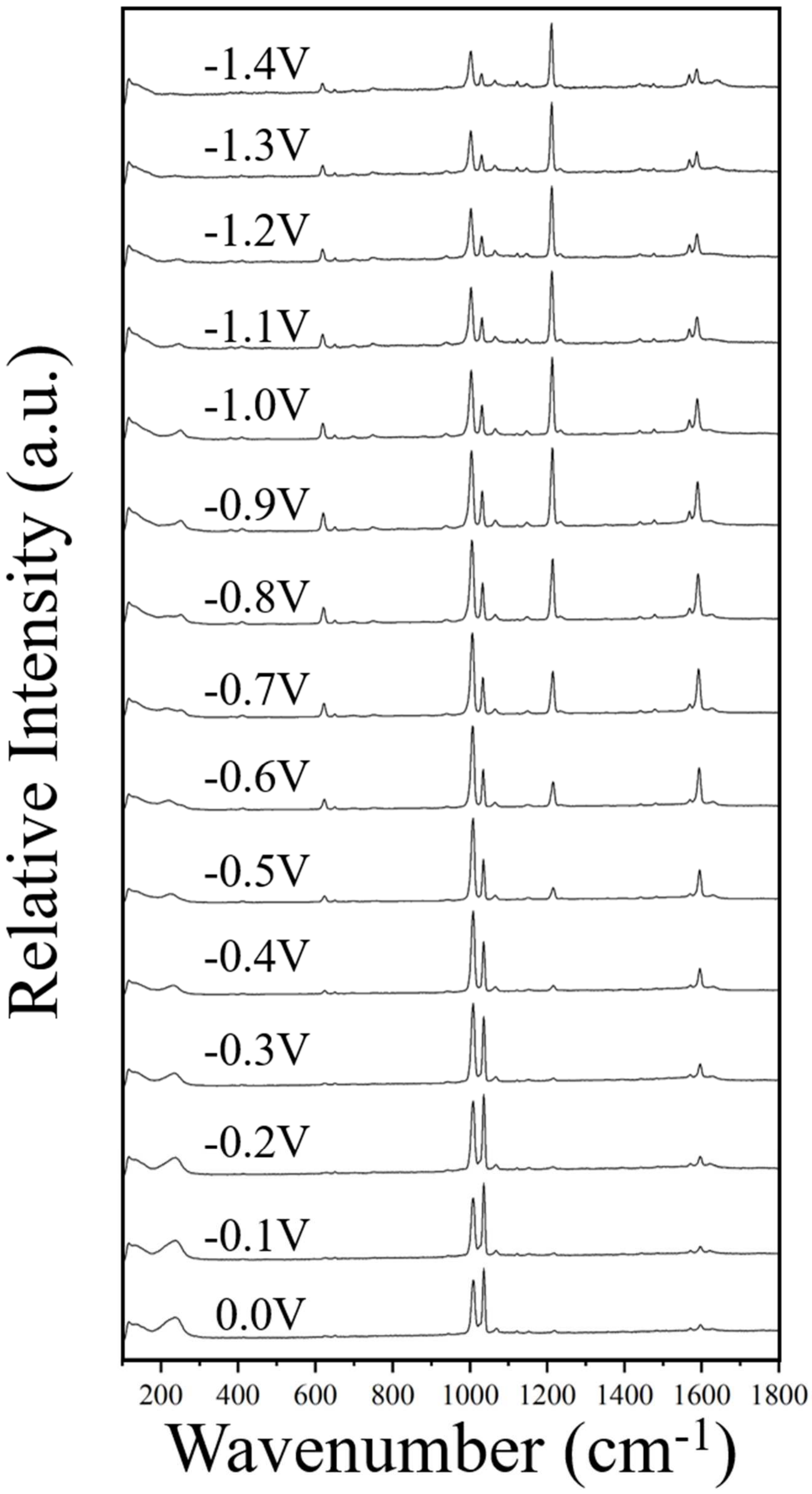

Supplementary Figure S1 Potential-dependent SERS spectra of Py aqueous solution (0.1 M) on silver electrode recorded in steps of 0.1 V. Excitation wavelength = 514 nm.

Supplementary Table S1 Absolute vibrational SERS wavenumbers (cm^{-1}) for the main vibrations of aqueous pyridine (0.1 M) recorded on silver at different electrode potentials.

SERS (V)															
0.0	-0.1	-0.2	-0.3	-0.4	-0.5	-0.6	-0.7	-0.8	-0.9	-1.0	-1.1	-1.2	-1.3	-1.4	Mode
1596.7	1596.1	1596.0	1596.1	1595.4	1594.0	1593.7	1591.8	1590.7	1589.5	1588.6	1588.0	1587.7	1587.1	1586.6	8a
1571.1	1570.7	1570.7	1570.7	1570.7	1570.7	1569.9	1569.2	1569.2	1568.7	1567.7	1567.7	1567.7	1567.7	1567.7	8b
-	-	-	-	-	-	1481.5	1479.0	1478.3	1478.0	1477.5	1477.5	1477.5	1476.0	1476.0	19a
1219.0	1216.4	1216.4	1216.2	1216.0	1215.7	1215.4	1214.8	1214.1	1213.8	1212.6	1212.3	1211.7	1211.7	1211.0	9a
1069.4	1068.5	1068.5	1067.7	1066.9	1066.9	1066.9	1066.9	1066.9	1066.9	1066.9	1066.9	1065.8	1065.3	1065.3	18a
1036.2	1036.2	1036.2	1036.0	1035.9	1035.0	1034.6	1034.2	1033.1	1032.1	1031.3	1031.0	1030.9	1030.3	1030.3	12
1008.4	1007.9	1007.9	1007.9	1007.9	1007.5	1006.9	1005.8	1005.2	1003.8	1003.1	1002.5	1002.2	1001.5	1001.4	1
650.4	650.4	650.4	650.4	650.4	650.4	650.4	650.4	650.4	650.4	650.4	650.4	650.4	650.4	650.4	6b
625.3	625.3	625.3	625.3	624.1	623.7	623.0	622.0	621.0	620.3	619.5	618.7	618.7	618.7	618.7	6a

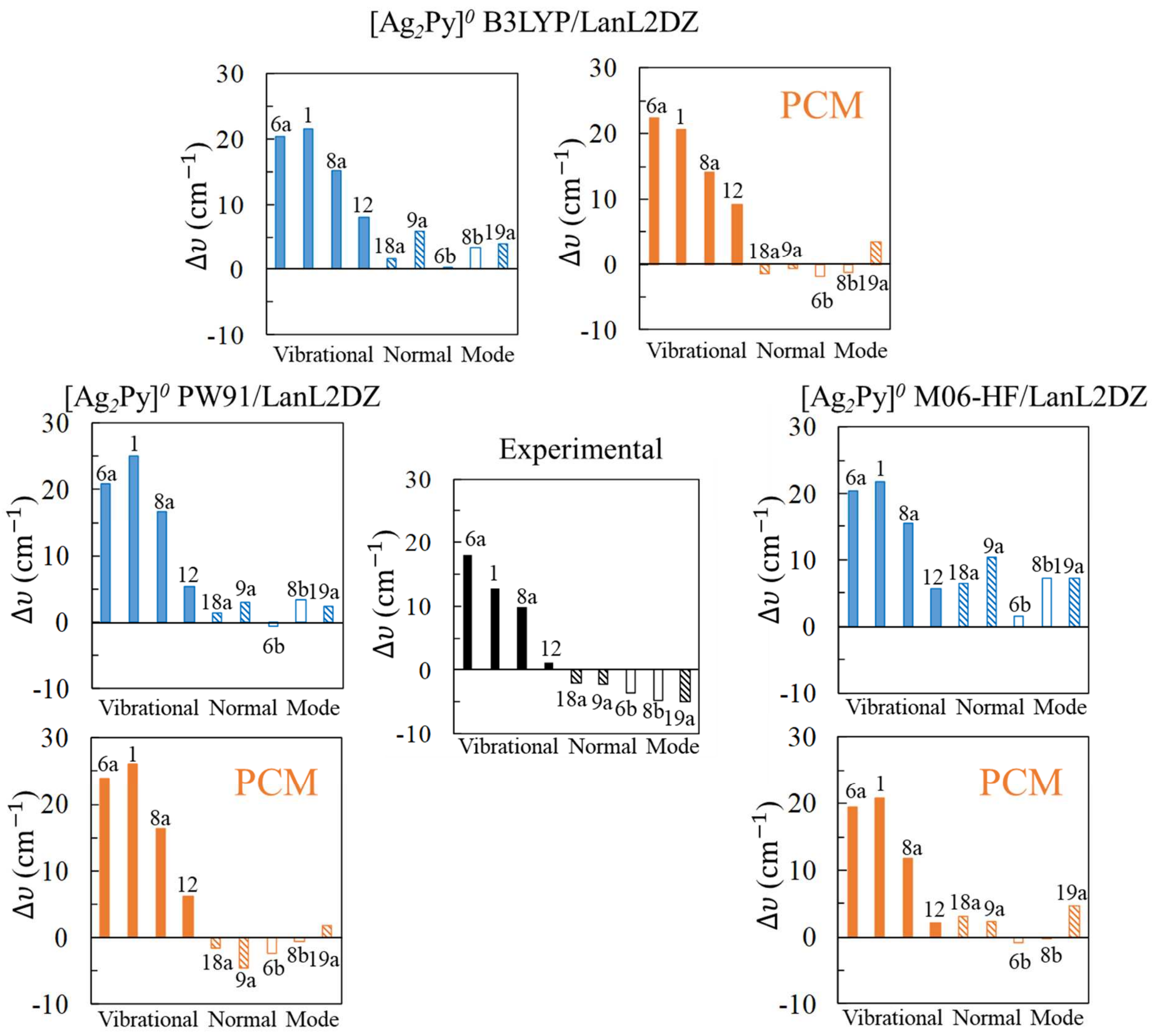

Supplementary Figure S2 Experimental (SERS at -0.7 V) wavenumber shifts $\Delta\upsilon$ and calculated values at B3LYP, PW91 and M06-HF/LanL2DZ levels of theory from isolated and solvated (PCM) $[Ag_2Py]^0$ complex.

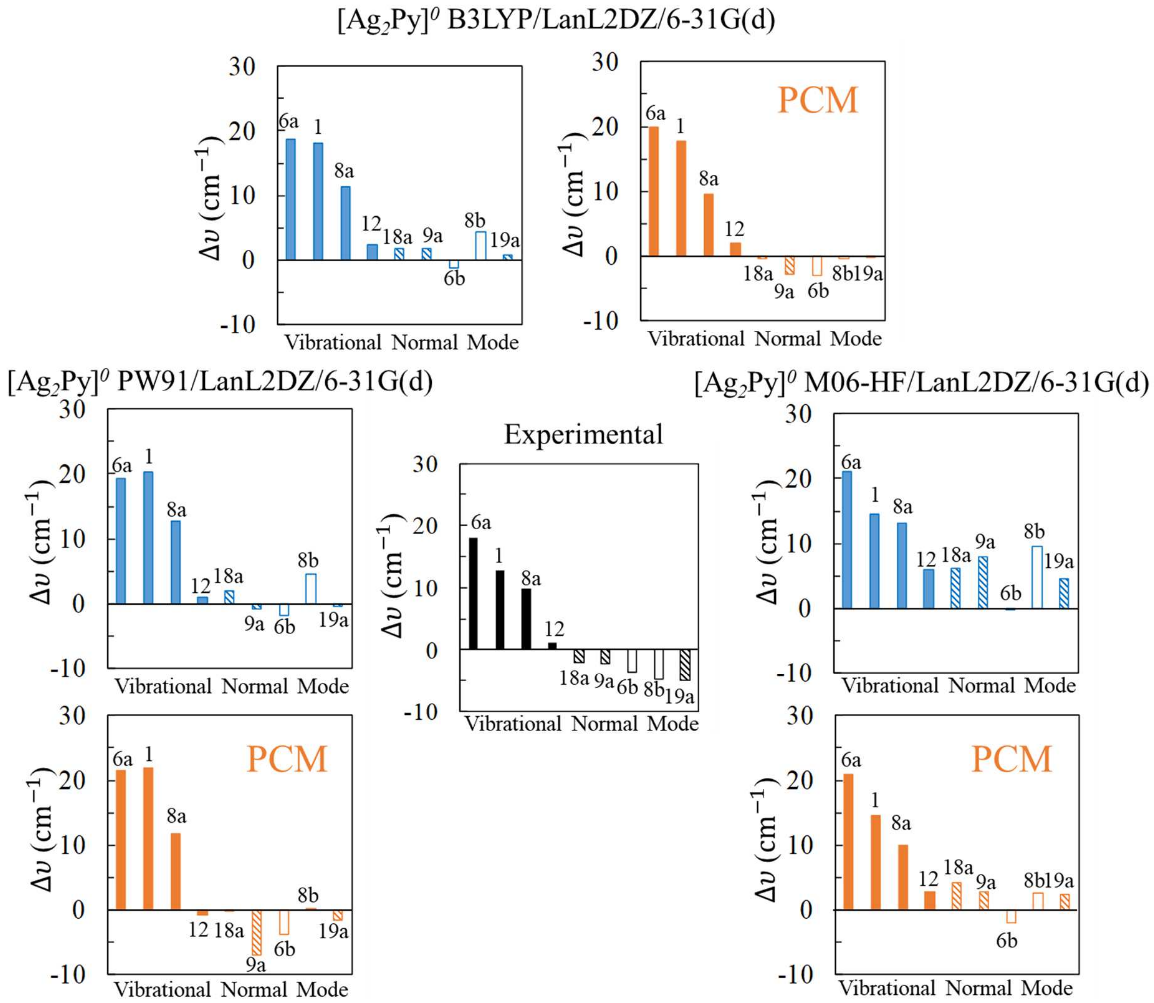

Supplementary Figure S3 Experimental (SERS at -0.7 V) wavenumber shifts $\Delta\upsilon$ and calculated values at B3LYP, PW91 and M06-HF/LanL2DZ/6-31G(d) levels of theory from isolated and solvated (PCM) $[Ag_2Py]^0$ complex.

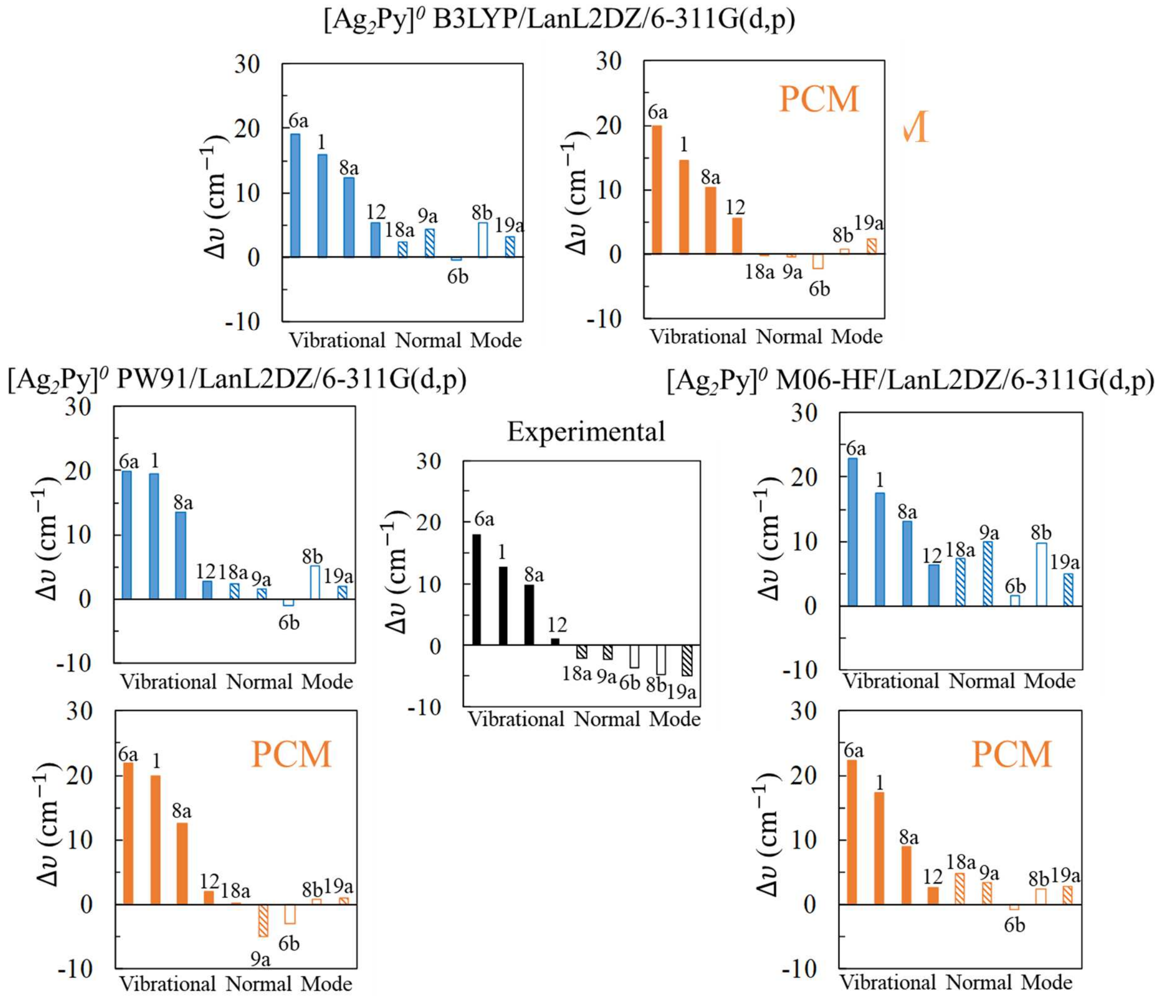

Supplementary Figure S4 Experimental (SERS at -0.7 V) wavenumber shifts $\Delta\upsilon$ and calculated values at B3LYP, PW91 and M06-HF/LanL2DZ/6-311G(d,p) levels of theory from isolated and solvated (PCM) $[Ag_2Py]^0$ complex.

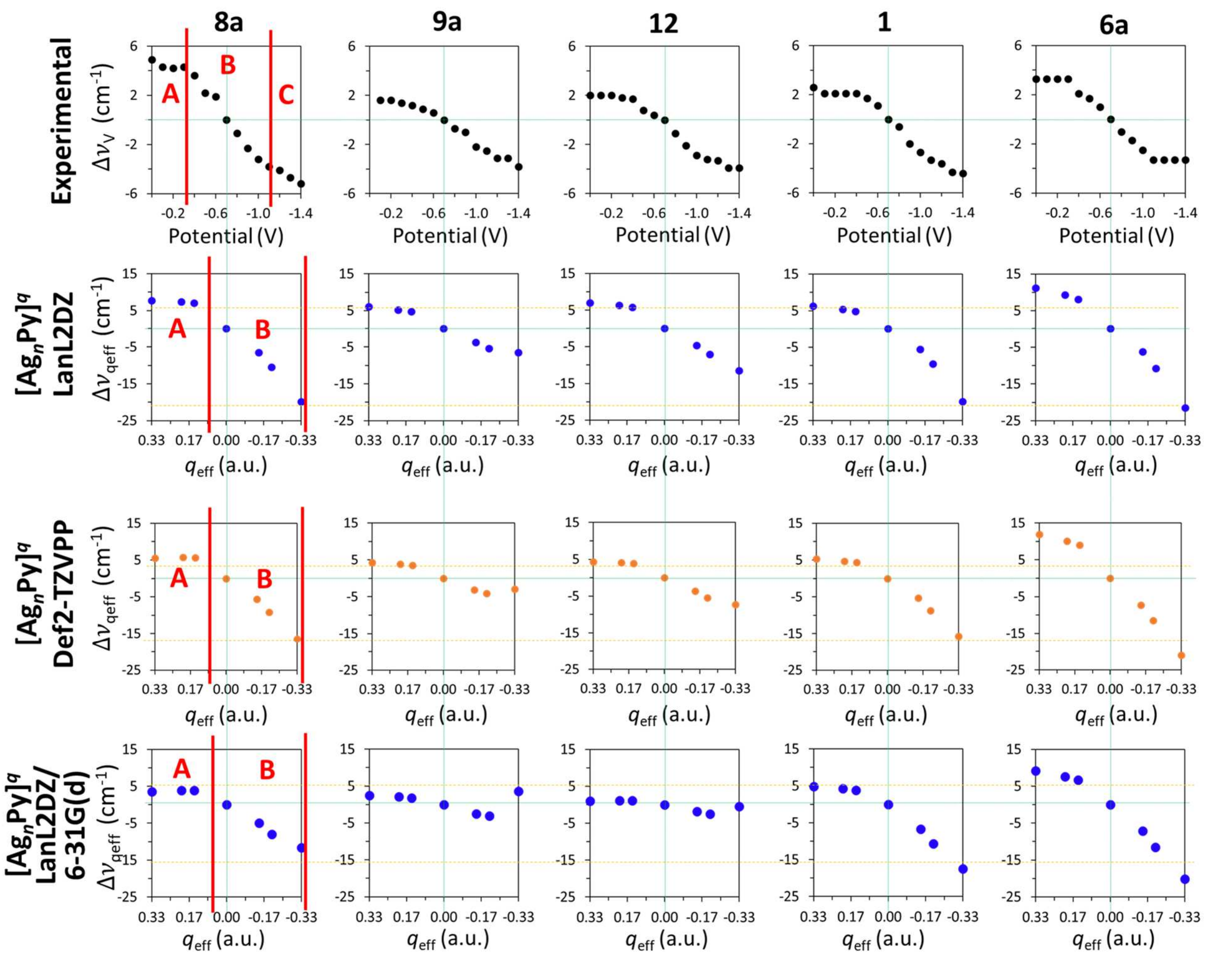

Supplementary Figure S5 Tuning of the experimental wavenumbers of pyridine vibrations by the electrode potential (top, black) and of the B3LYP/LanL2DZ (middle, blue), B3LYP/def2-TZVPP (midle, orange) or B3LYP/LanL2DZ/6-31G(d) (bottom, blue) calculated wavenumbers by the effective charge of isolated $[Ag_nPy]^q$ complexes.

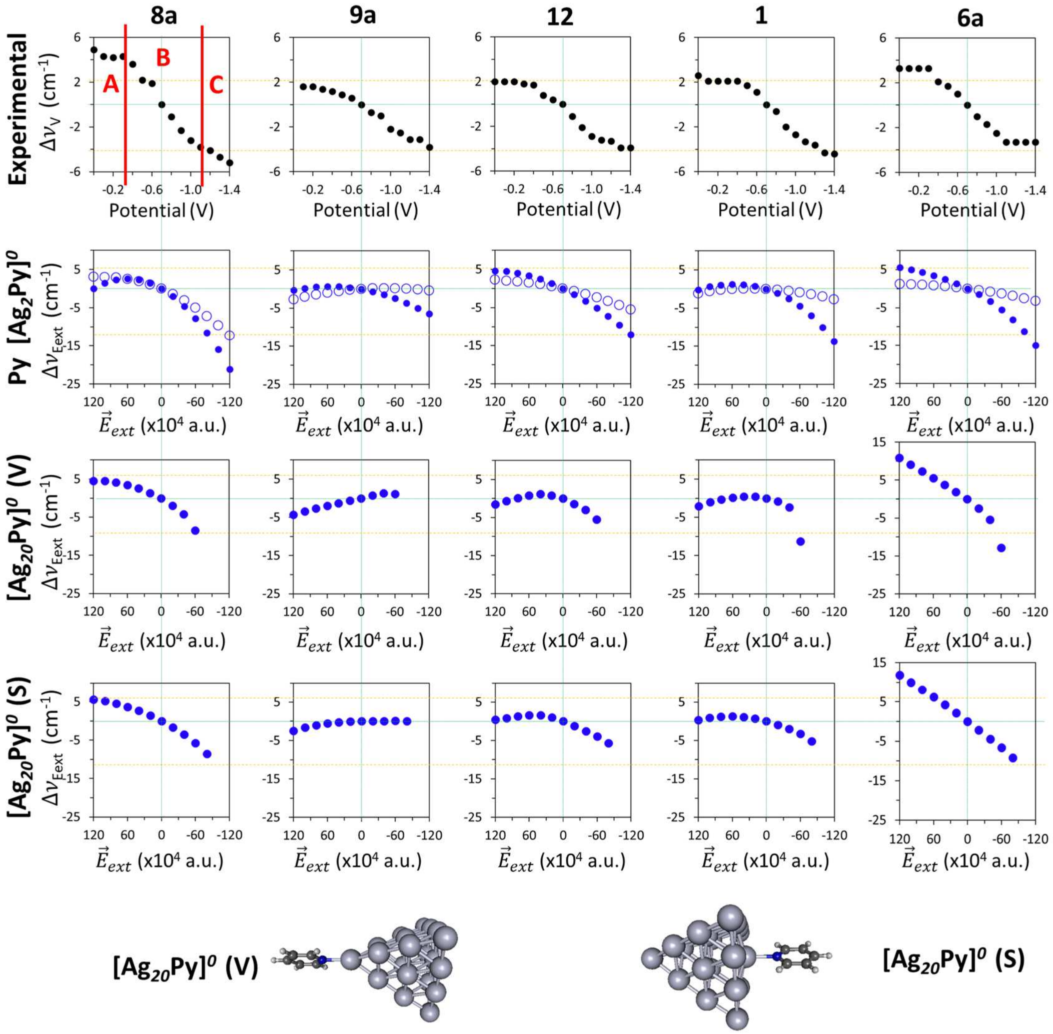

Supplementary Figure S6 Tuning of the experimental wavenumbers of pyridine vibrations on the electrode potential and of the B3LYP/LanL2DZ calculated wavenumbers on external electric fields of isolated Py (empty circles) and of $[Ag_2Py]^0$, $[Ag_{20}Py]^0$ (V) and $[Ag_{20}Py]^0$ (S) complexes (full circles).

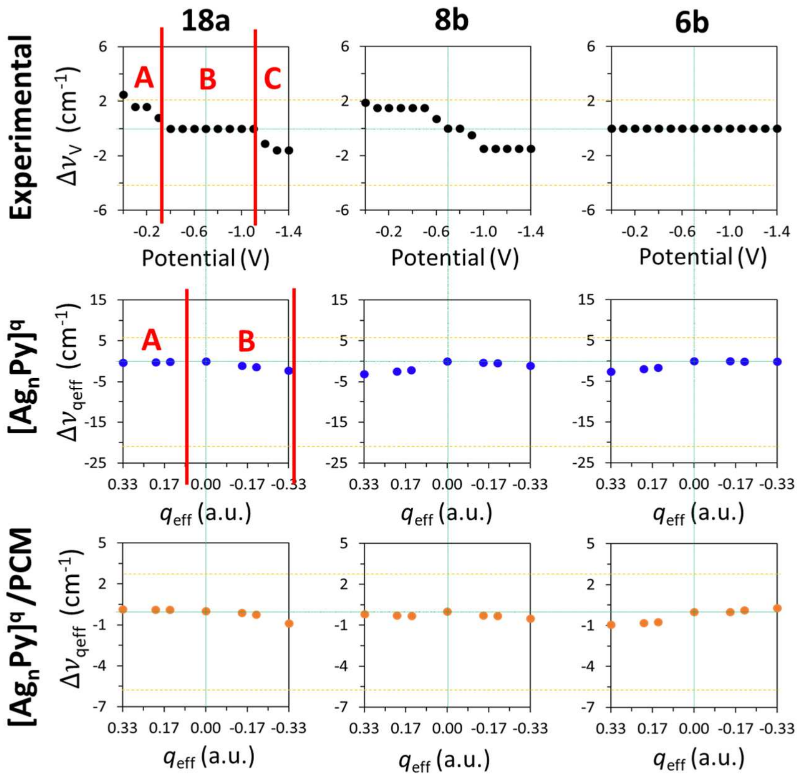

Supplementary Figure S7 Tuning of the experimental wavenumbers of pyridine vibrations by the electrode potential (top) and of the B3LYP/LanL2DZ calculated wavenumbers by the effective charge of isolated $[Ag_nPy]^q$ complexes (middle) or in a PCM environment (bottom).

$[Ag_nPy]^q$ B3LYP/LanL2DZ

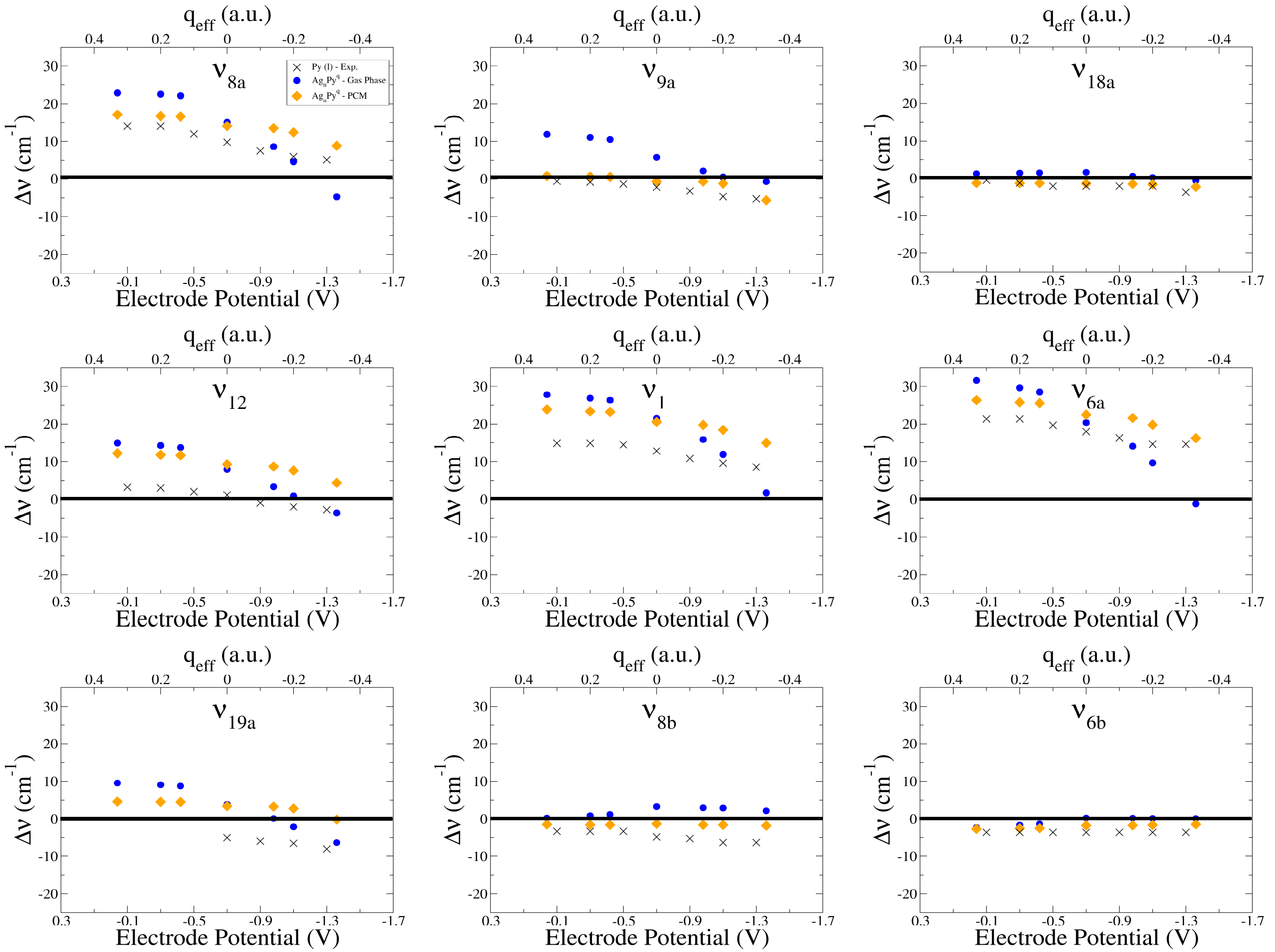

Supplementary Figure S8 Experimental wavenumber shifts $\Delta\upsilon$ (crosses) and B3LYP/LanL2DZ calculated values $\Delta\upsilon$ from isolated (blue) and solvated (PCM, orange) $[Ag_nPy]^q$ complexes.

$[Ag_nPy]^q$ PW91/LanL2DZ

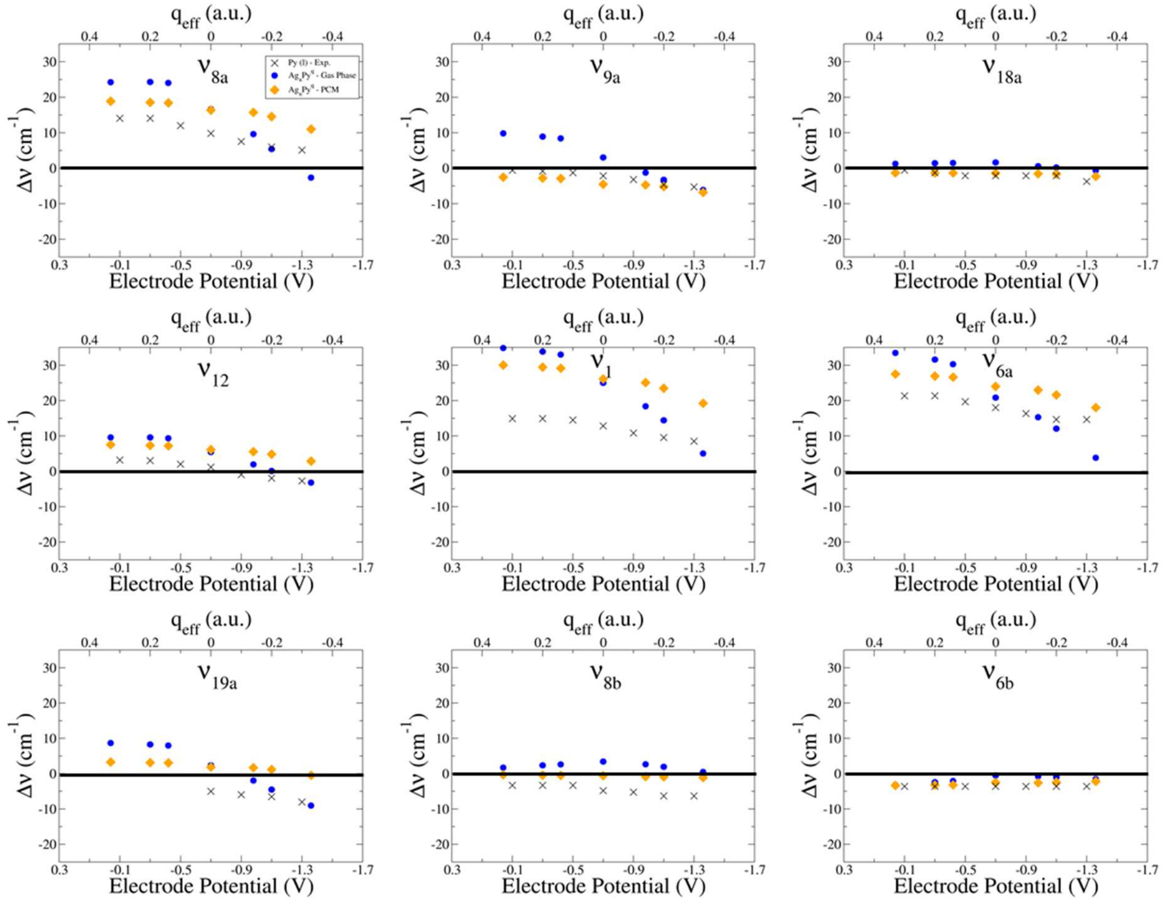

Supplementary Figure S9 Experimental wavenumber shifts $\Delta\upsilon$ (crosses) and PW91/LanL2DZ calculated values $\Delta\upsilon$ from isolated (blue) and solvated (PCM, orange) $[Ag_nPy]^q$ complexes.

$[Ag_nPy]^q$ M06-HF/LanL2DZ

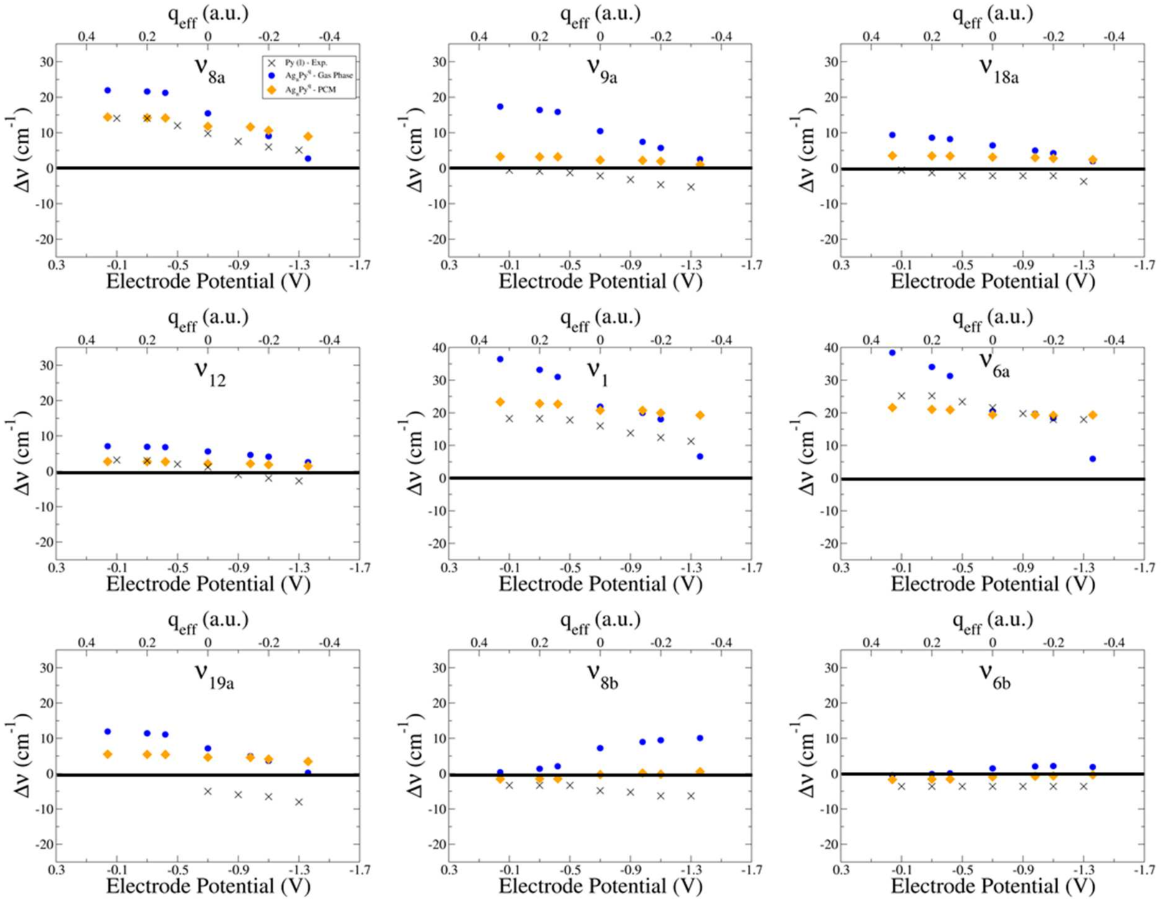

Supplementary Figure S10 Experimental wavenumber shifts $\Delta\upsilon$ (crosses) and M06-HF/LanL2DZ calculated values $\Delta\upsilon$ from isolated (blue) and solvated (PCM, orange) $[Ag_nPy]^q$ complexes.

$[Ag_nPy]^q$ B3LYP/LanL2DZ/6-31G(d)

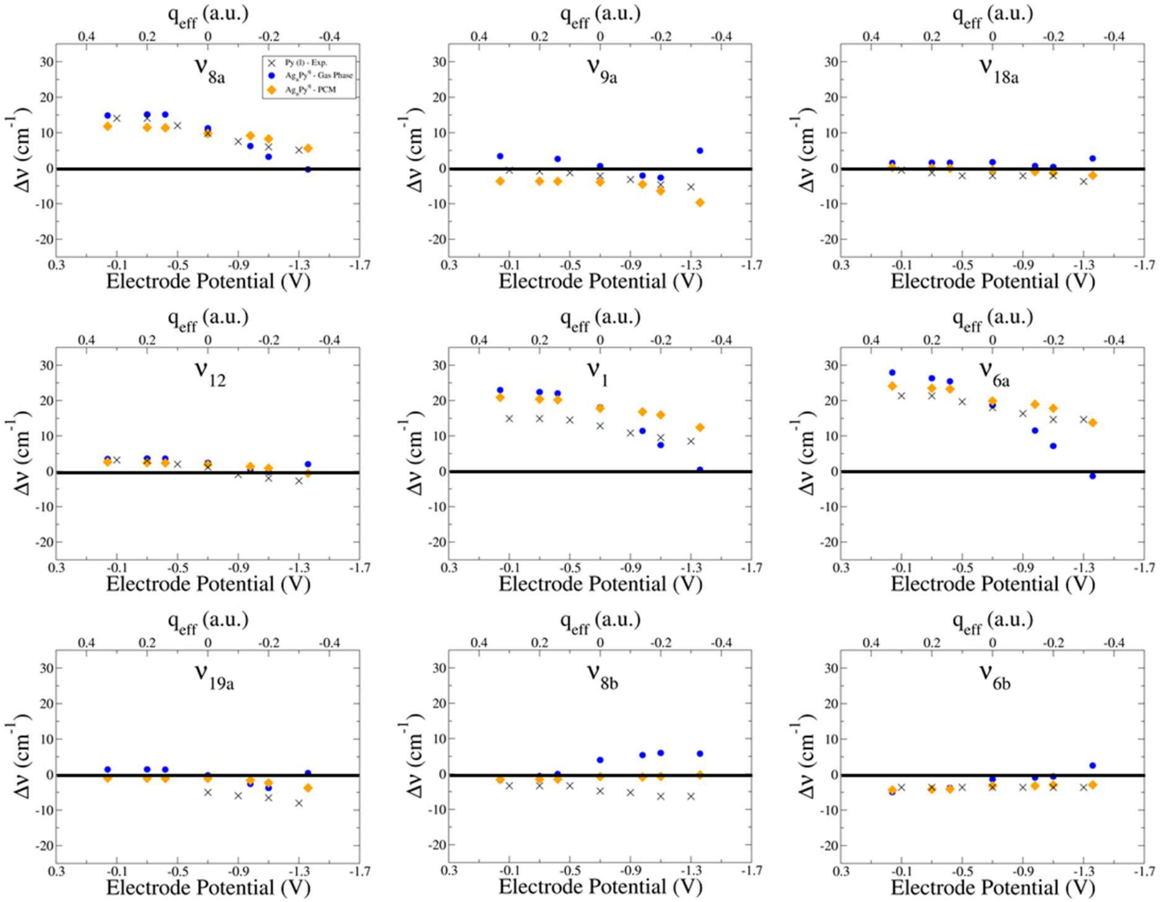

Supplementary Figure S11 Experimental wavenumber shifts $\Delta\upsilon$ (crosses) and B3LYP/LanL2DZ/6-31G(d) calculated values $\Delta\upsilon$ from isolated (blue) and solvated (PCM, orange) $[Ag_nPy]^q$ complexes.

$[Ag_nPy]^q$ PW91/LanL2DZ/6-31G(d)

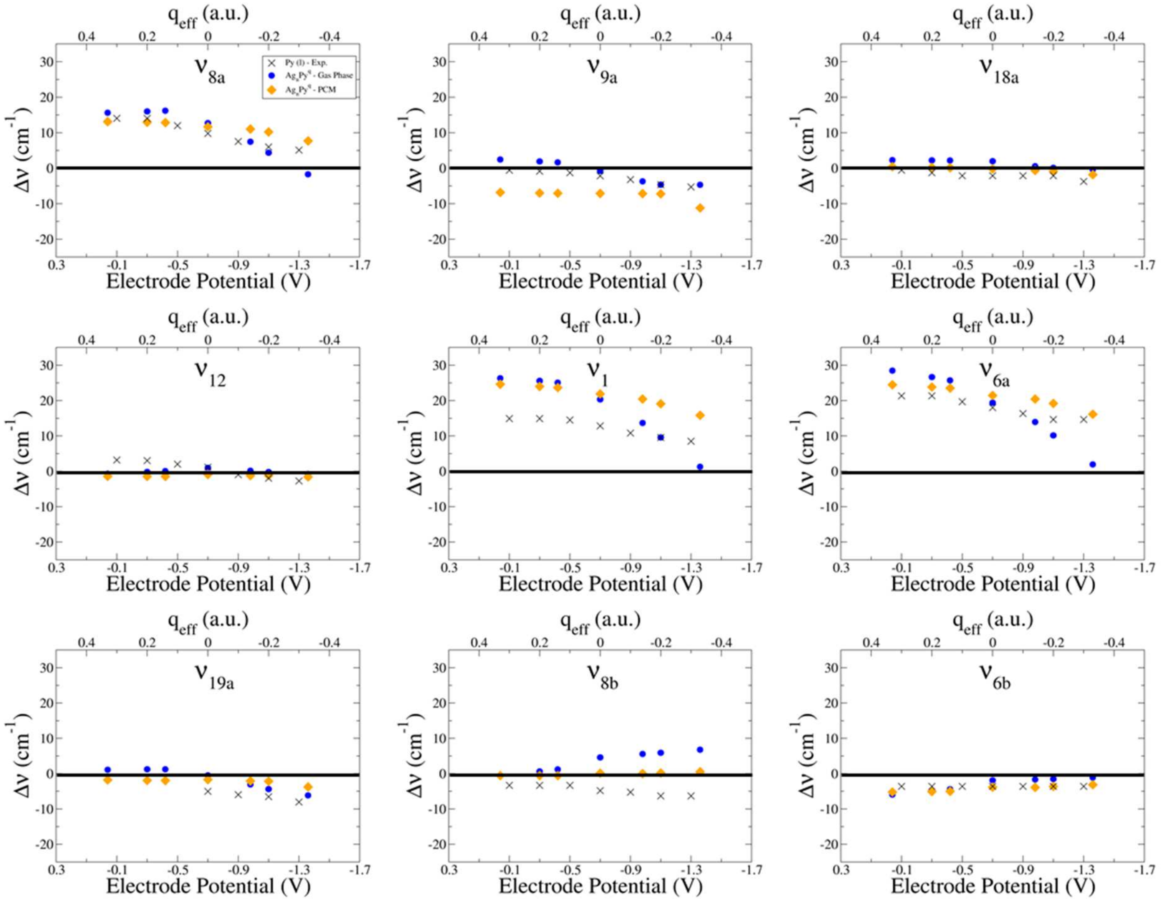

Supplementary Figure S12 Experimental wavenumber shifts $\Delta\upsilon$ (crosses) and PW91/LanL2DZ/6-31G(d) calculated values $\Delta\upsilon$ from isolated (blue) and solvated (PCM, orange) $[Ag_nPy]^q$ complexes.

$[Ag_nPy]^q$ M06-HF/LanL2DZ/6-31G(d)

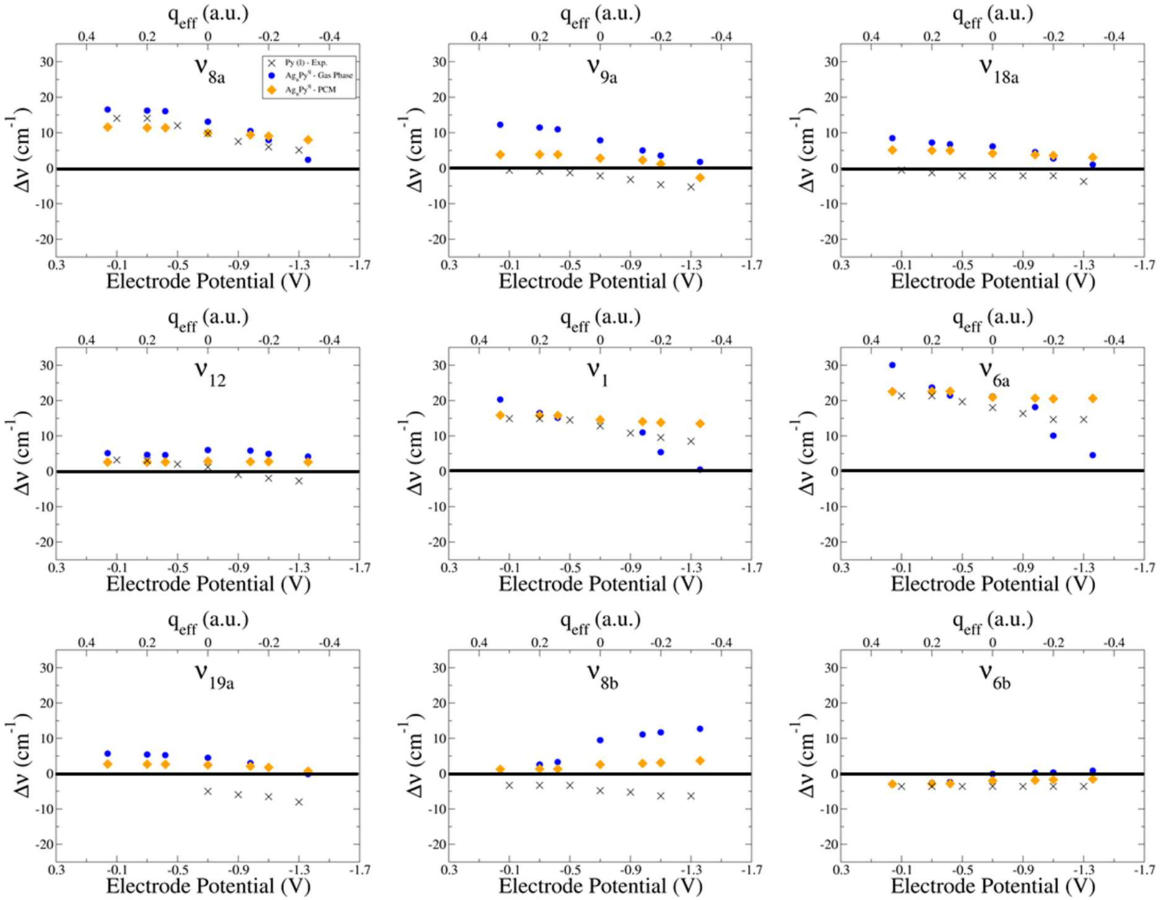

Supplementary Figure S13 Experimental wavenumber shifts $\Delta\upsilon$ (crosses) and M06-HF/LanL2DZ/6-31G(d) calculated values $\Delta\upsilon$ from isolated (blue) and solvated (PCM, orange) $[Ag_nPy]^q$ complexes.

$[Ag_nPy]^q$ B3LYP/LanL2DZ/6-311G(d,p)

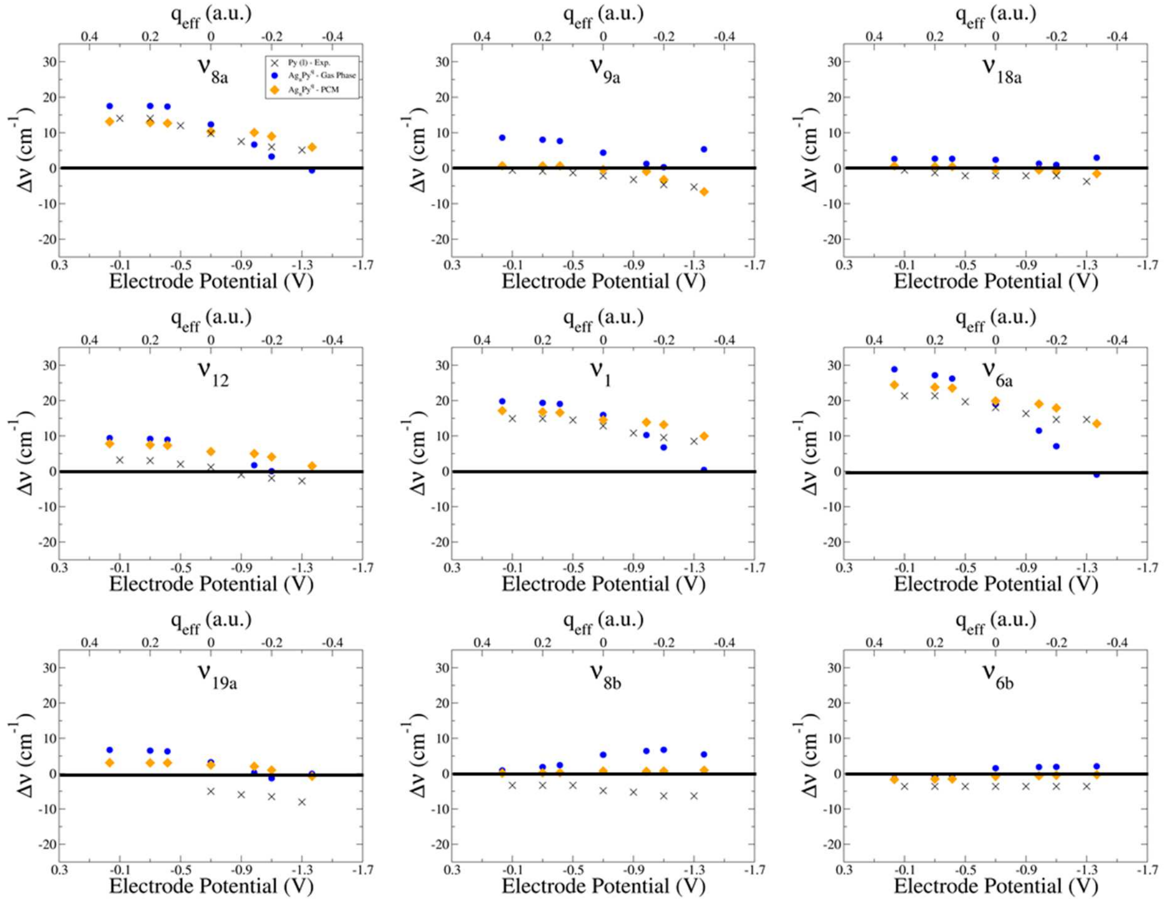

Supplementary Figure S14 Experimental wavenumber shifts $\Delta\upsilon$ (crosses) and B3LYP/LanL2DZ/6-311G(d,p) calculated values $\Delta\upsilon$ from isolated (blue) and solvated (PCM, orange) $[Ag_nPy]^q$ complexes.

$[Ag_nPy]^q$ PW91/LanL2DZ/6-311G(d,p)

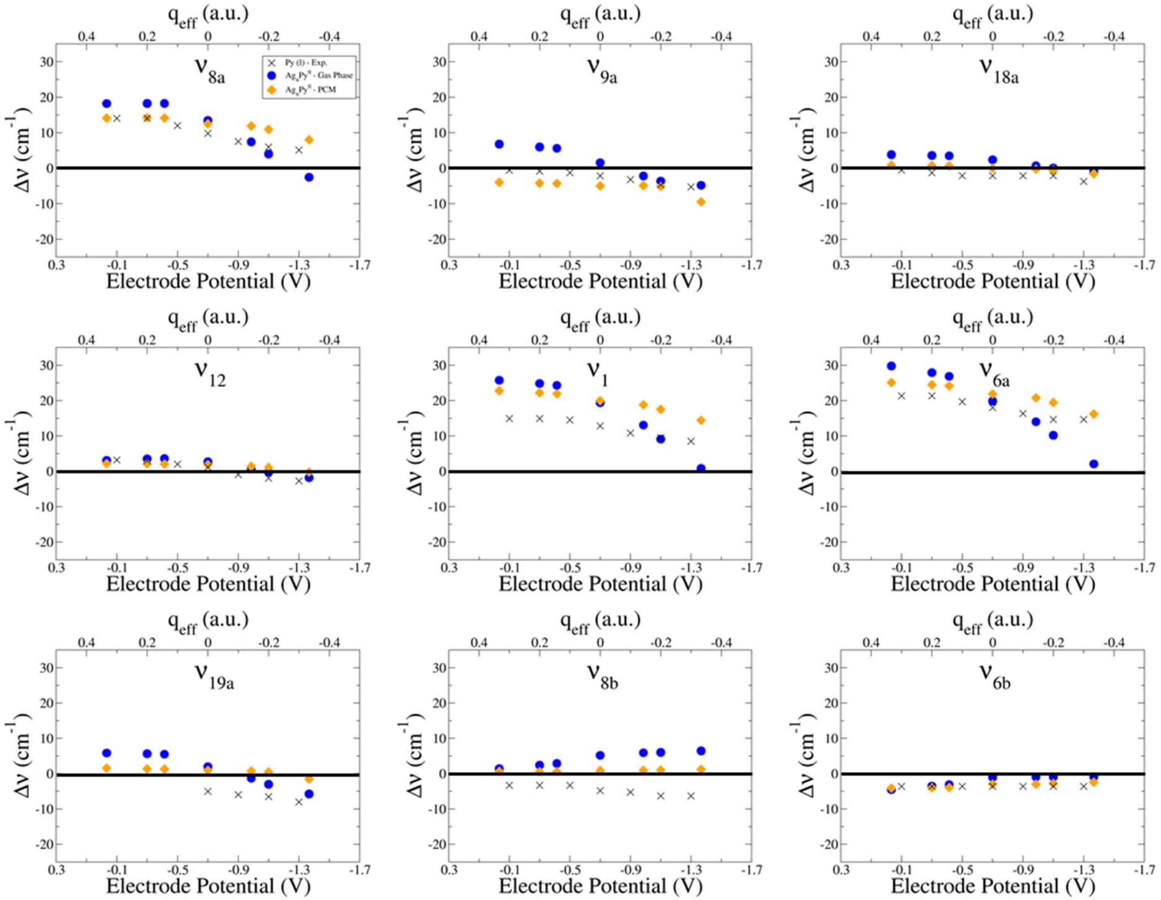

Supplementary Figure S15 Experimental wavenumber shifts $\Delta\upsilon$ (crosses) and PW91/LanL2DZ/6-311G(d,p) calculated values $\Delta\upsilon$ from isolated (blue) and solvated (PCM, orange) $[Ag_nPy]^q$ complexes.

$[Ag_nPy]^q$ M06-HF/LanL2DZ/6-311G(d,p)

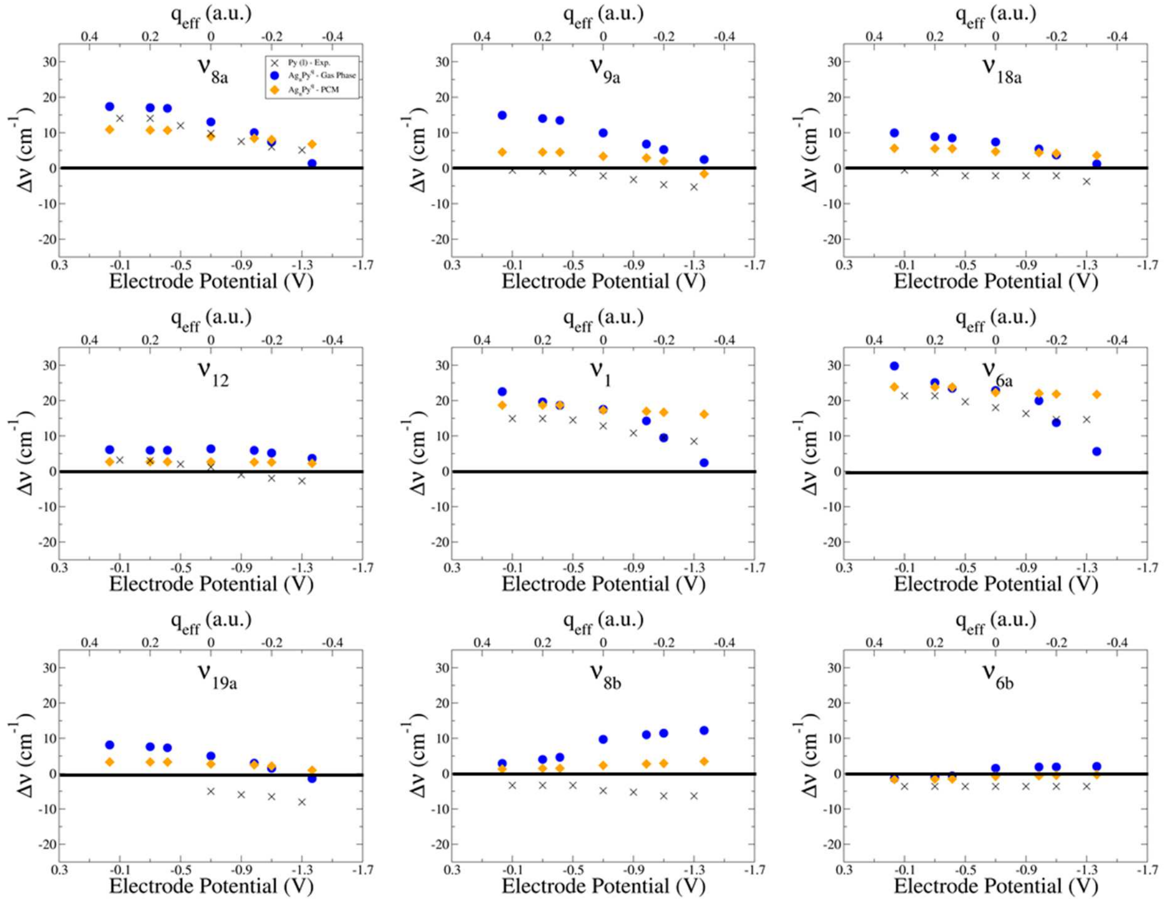

Supplementary Figure S16 Experimental wavenumber shifts $\Delta\upsilon$ (crosses) and M06-HF/LanL2DZ/6-311G(d,p) calculated values $\Delta\upsilon$ from isolated (blue) and solvated (PCM, orange) $[Ag_nPy]^q$ complexes.

Py B3LYP/LanL2DZ

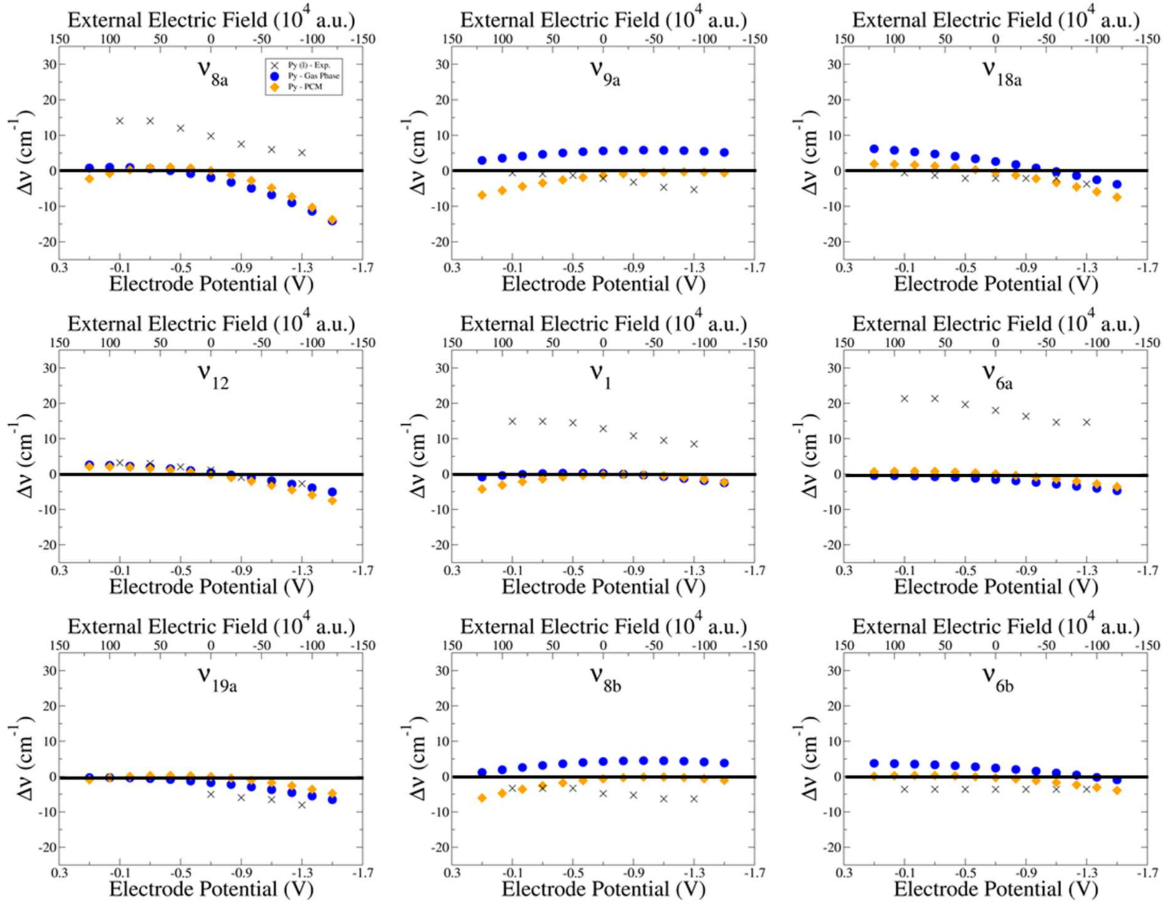

Supplementary Figure S17 Experimental wavenumber shifts $\Delta\upsilon$ (crosses) and B3LYP/LanL2DZ calculated values under an external electric field from isolated (blue) and solvated (PCM, orange) pyridine.

Py PW91/LanL2DZ

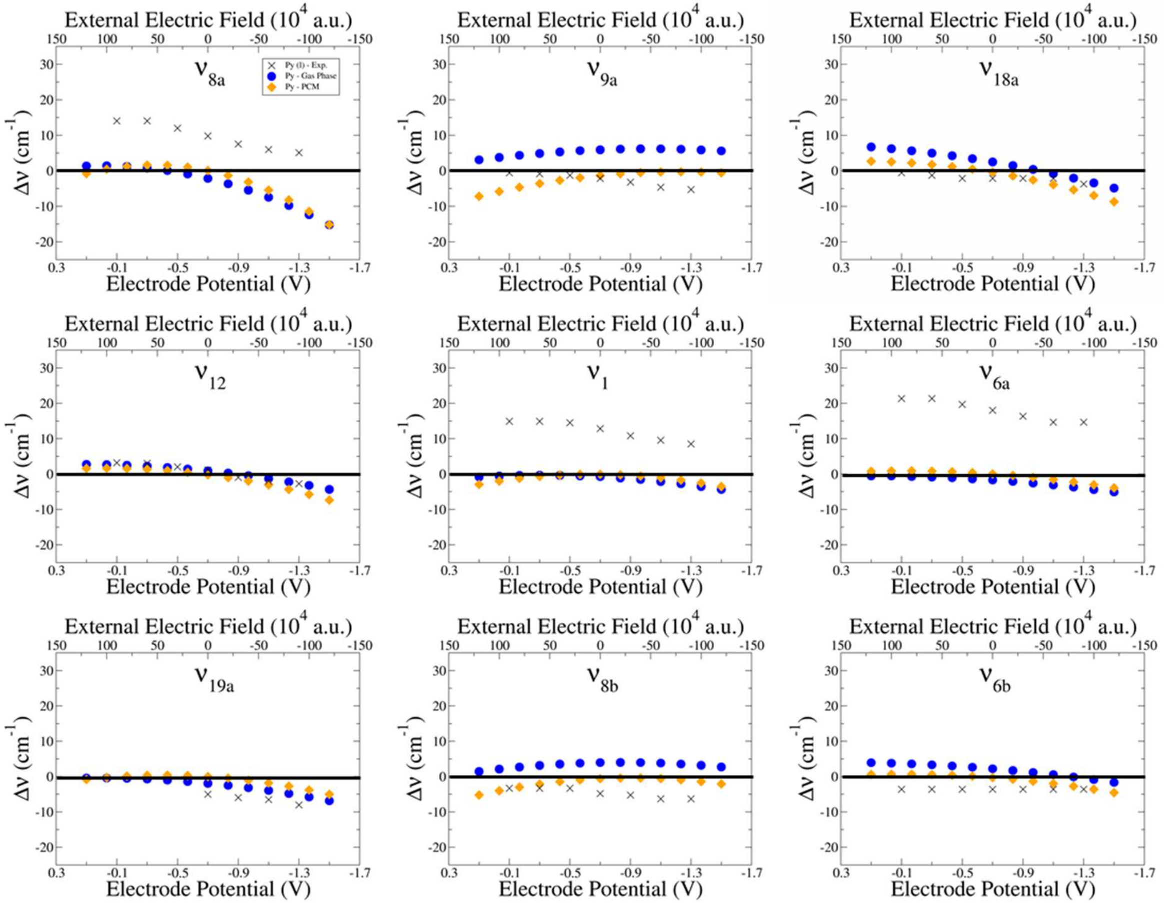

Supplementary Figure S18 Experimental wavenumber shifts $\Delta\upsilon$ (crosses) and PW91/LanL2DZ calculated values under an external electric field from isolated (blue) and solvated (PCM, orange) pyridine.

Py M06-HF/LanL2DZ

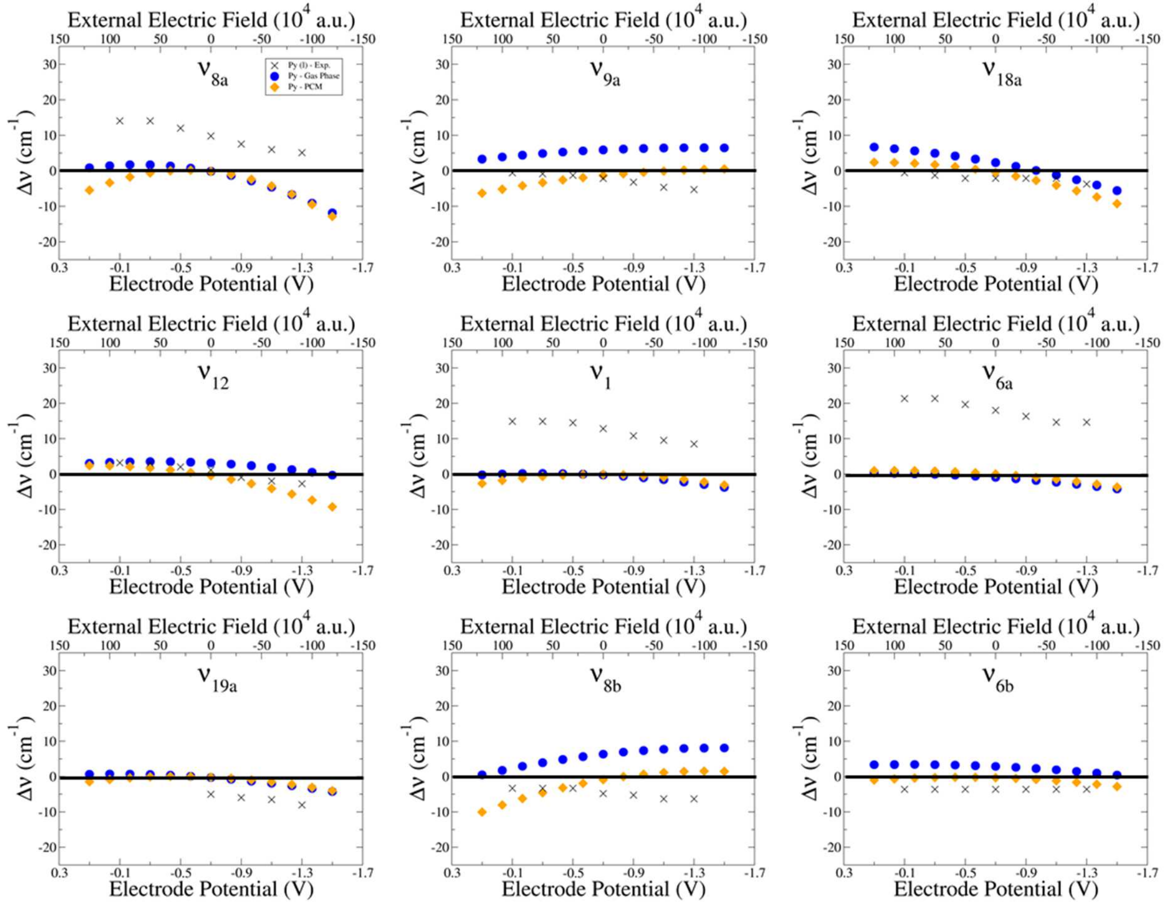

Supplementary Figure S19 Experimental wavenumber shifts $\Delta\upsilon$ (crosses) and M06-HF/LanL2DZ calculated values under an external electric field from isolated (blue) and solvated (PCM, orange) pyridine.

Py B3LYP/LanL2DZ/6-311G(d,p)

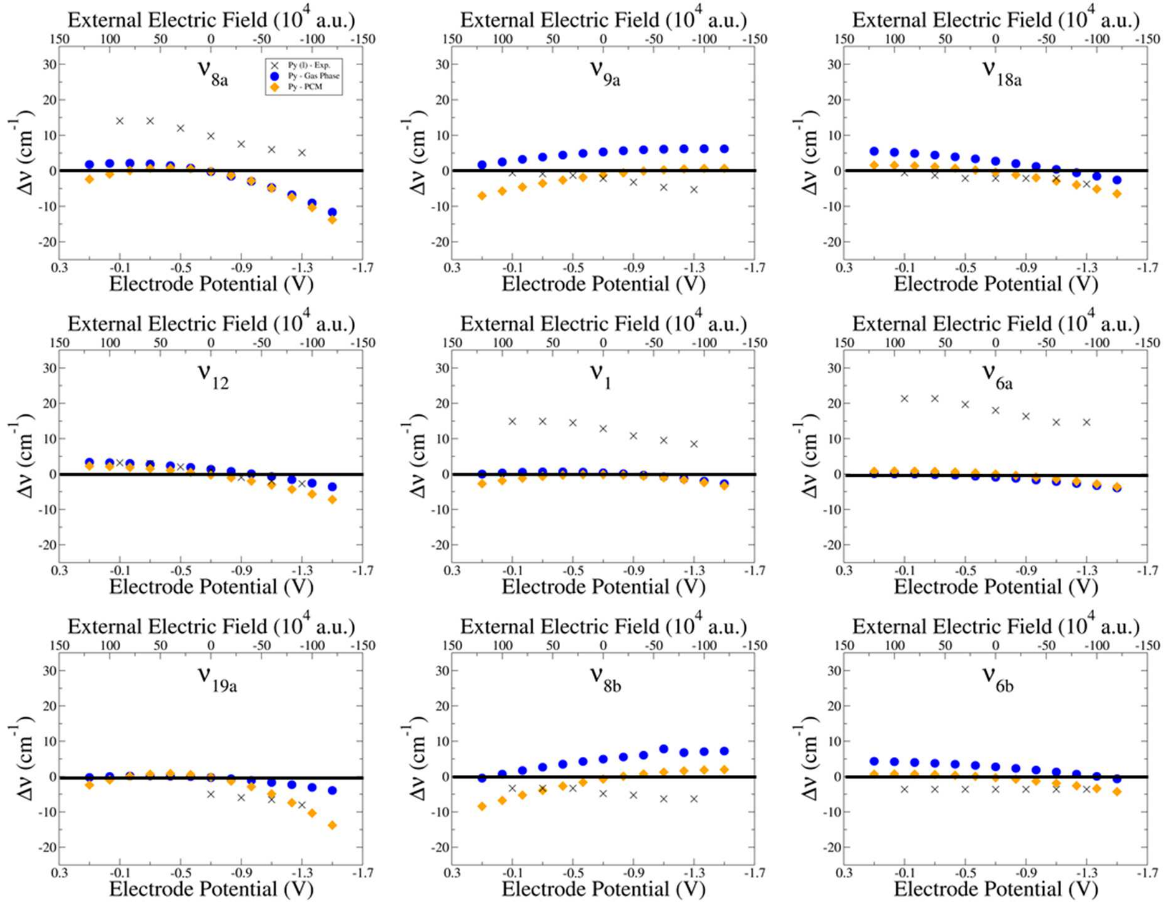

Supplementary Figure S20 Experimental wavenumber shifts $\Delta \upsilon$ (crosses) and B3LYP/LanL2DZ/6-311G(d,p) calculated values under an external electric field from isolated (blue) and solvated (PCM, orange) pyridine.

Py PW91/LanL2DZ/6-311G(d,p)

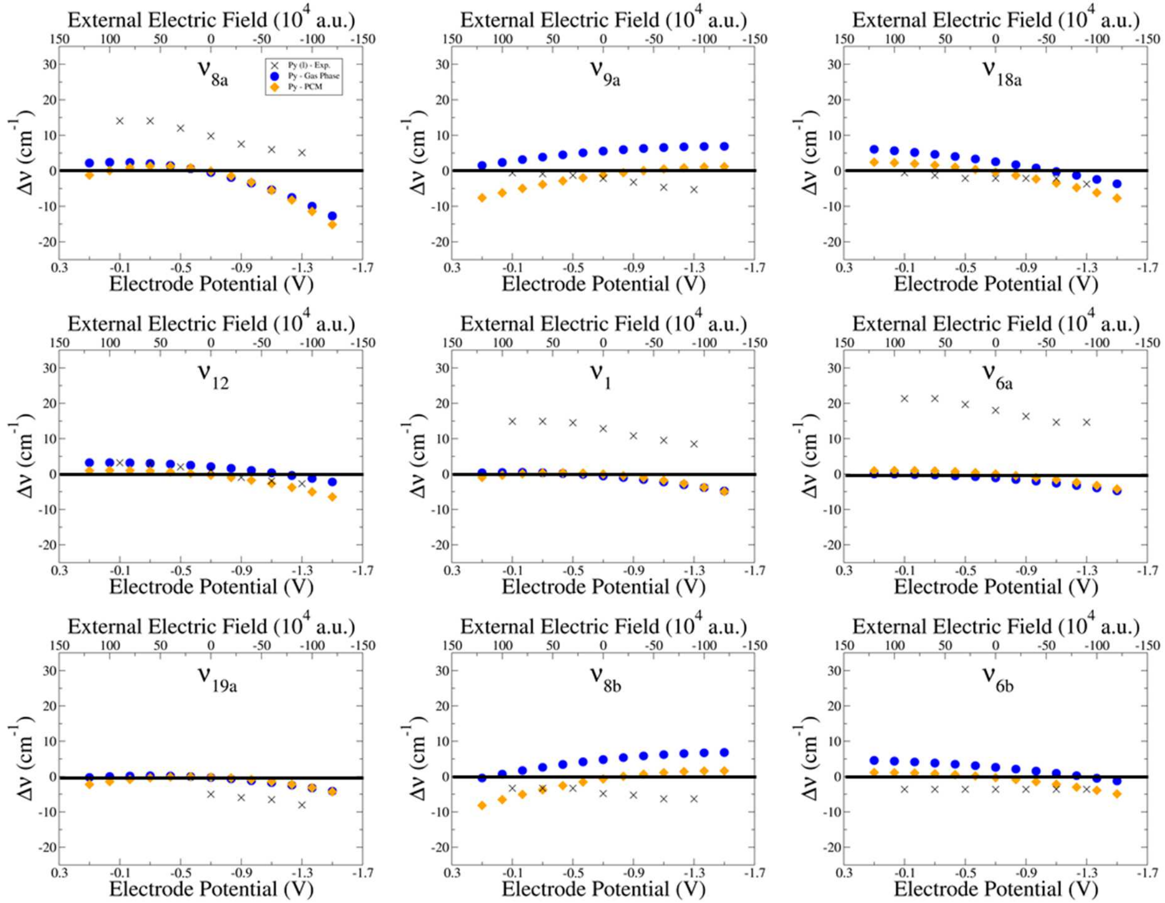

Supplementary Figure S21 Experimental wavenumber shifts $\Delta\upsilon$ (crosses) and PW91/LanL2DZ/6-311G(d,p) calculated values under an external electric field from isolated (blue) and solvated (PCM, orange) pyridine.

Py M06-HF/LanL2DZ/6-311G(d,p)

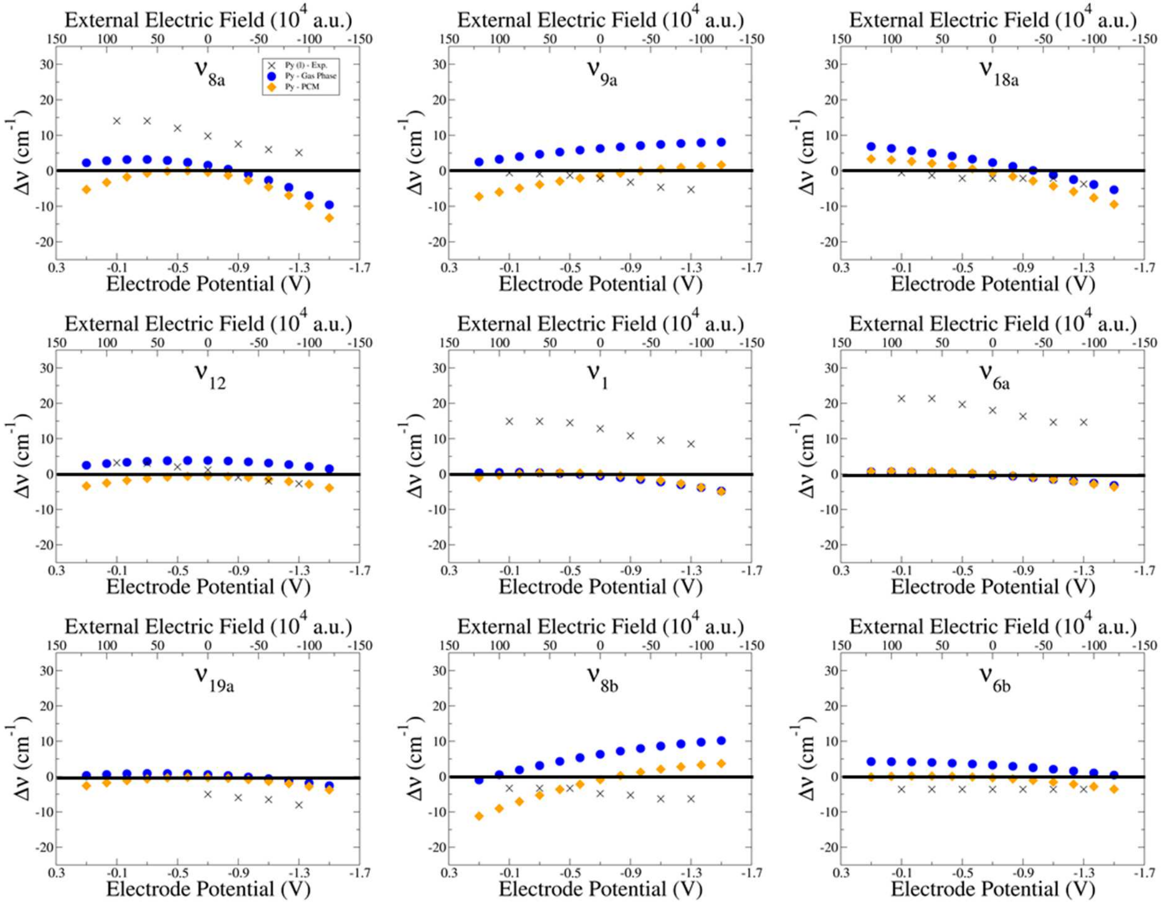

Supplementary Figure S22 Experimental wavenumber shifts $\Delta\upsilon$ (crosses) and M06-HF/LanL2DZ/6-311G(d,p) calculated values under an external electric field from isolated (blue) and solvated (PCM, orange) pyridine.

$[Ag_2Py]^0$ B3LYP/LanL2DZ

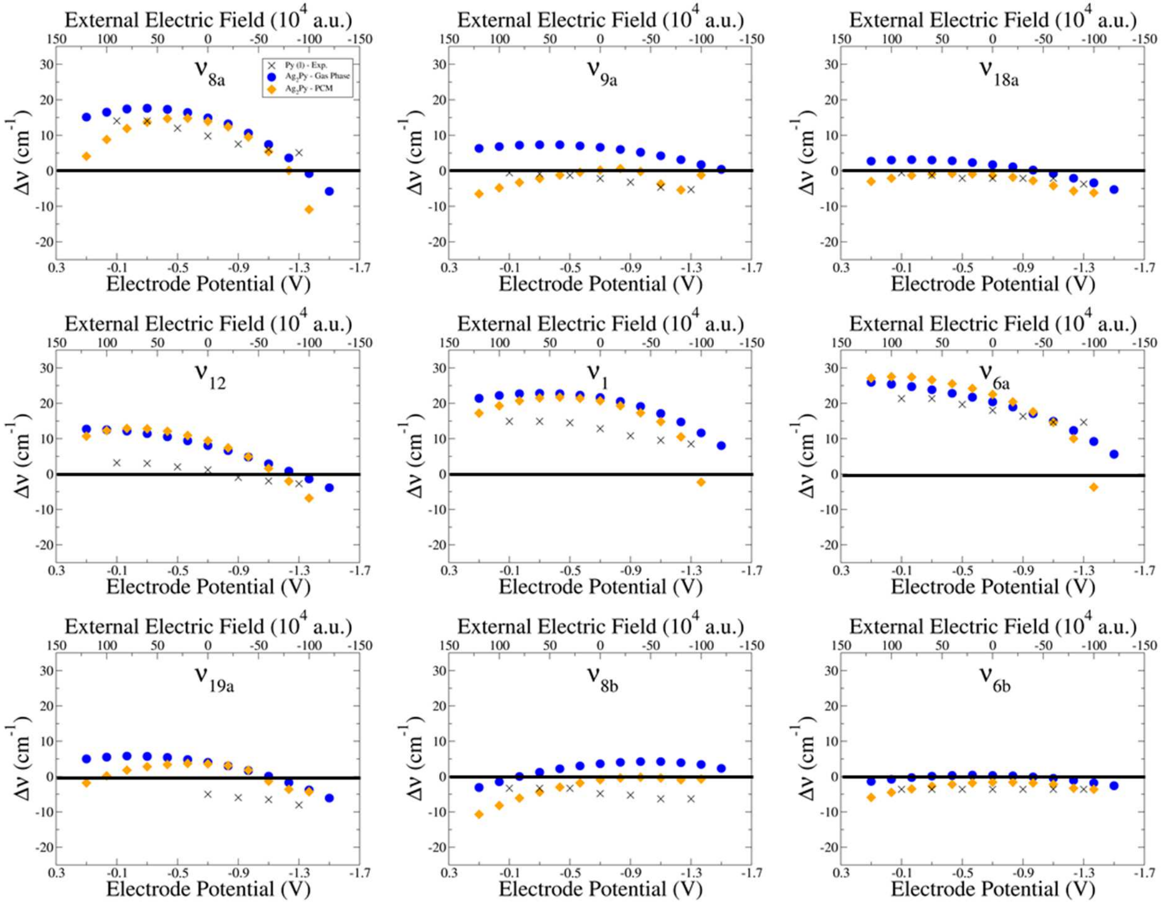

Supplementary Figure S23 Experimental wavenumber shifts $\Delta\upsilon$ (crosses) and B3LYP/LanL2DZ calculated values under an external electric field from isolated (blue) and solvated (PCM, orange) $[Ag_2Py]^0$ complex.

$[Ag_2Py]^0$ PW91/LanL2DZ

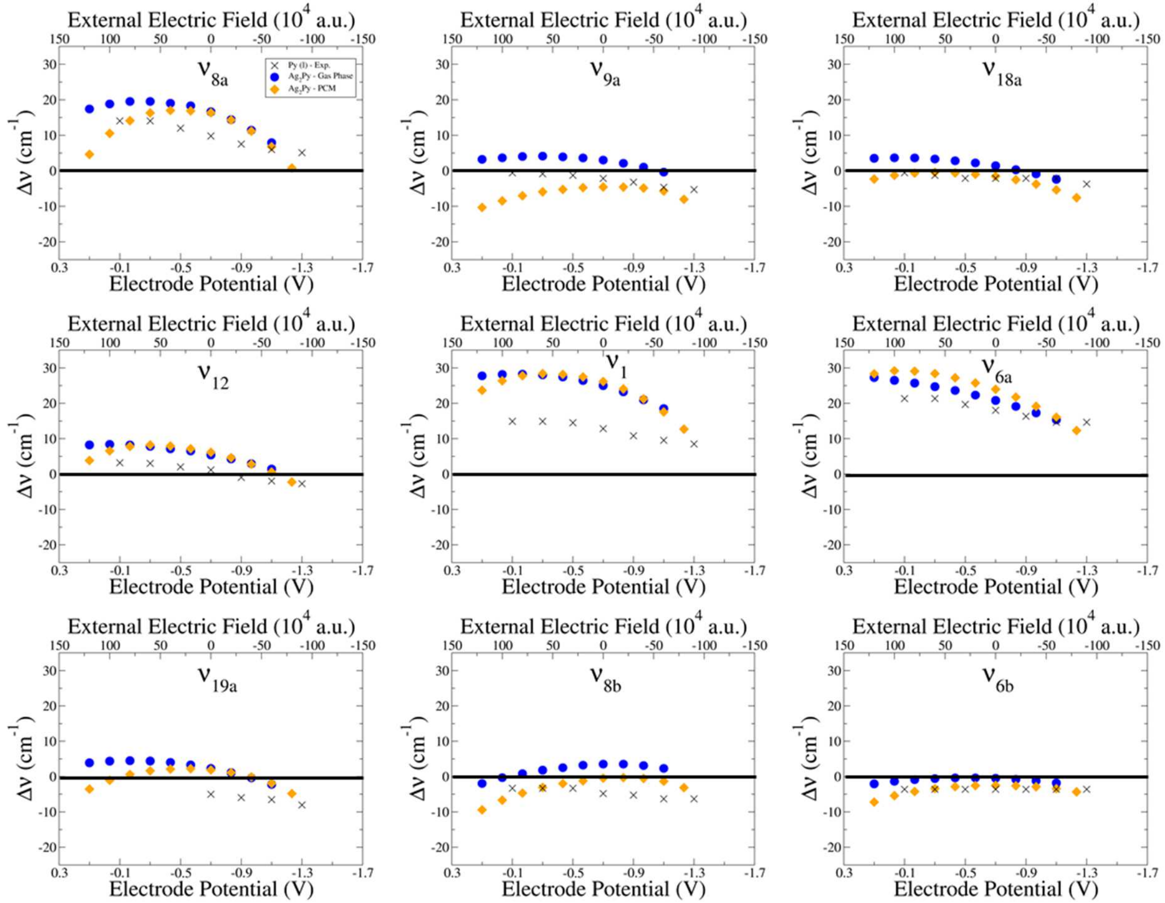

Supplementary Figure S24 Experimental wavenumber shifts $\Delta\upsilon$ (crosses) and PW91/LanL2DZ calculated values under an external electric field from isolated (blue) and solvated (PCM, orange) $[Ag_2Py]^0$ complex.

$[Ag_2Py]^0$ M06-HF/LanL2DZ

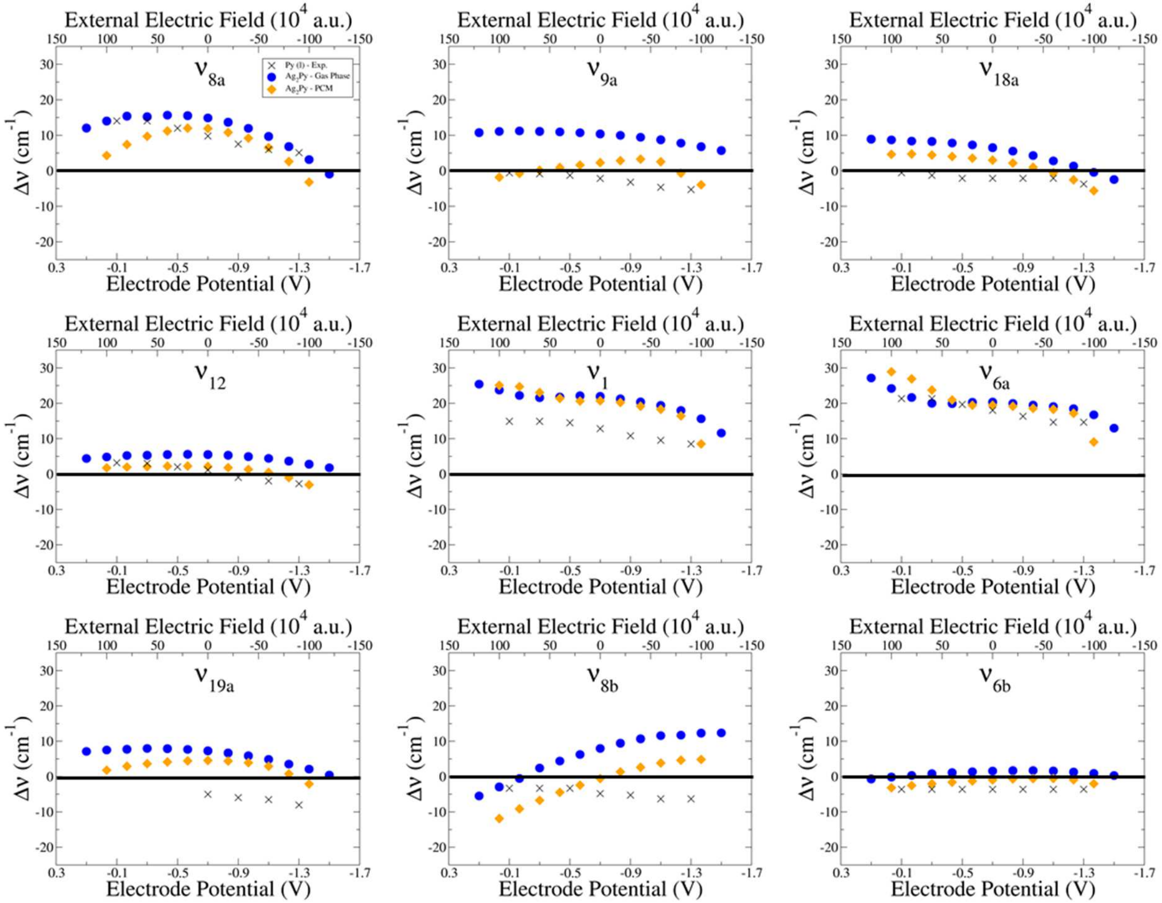

Supplementary Figure S25 Experimental wavenumber shifts $\Delta\upsilon$ (crosses) and M06-HF/LanL2DZ calculated values under an external electric field from isolated (blue) and solvated (PCM, orange) $[Ag_2Py]^0$ complex.

$[Ag_2Py]^0$ B3LYP/LanL2DZ/6-311G(d,p)

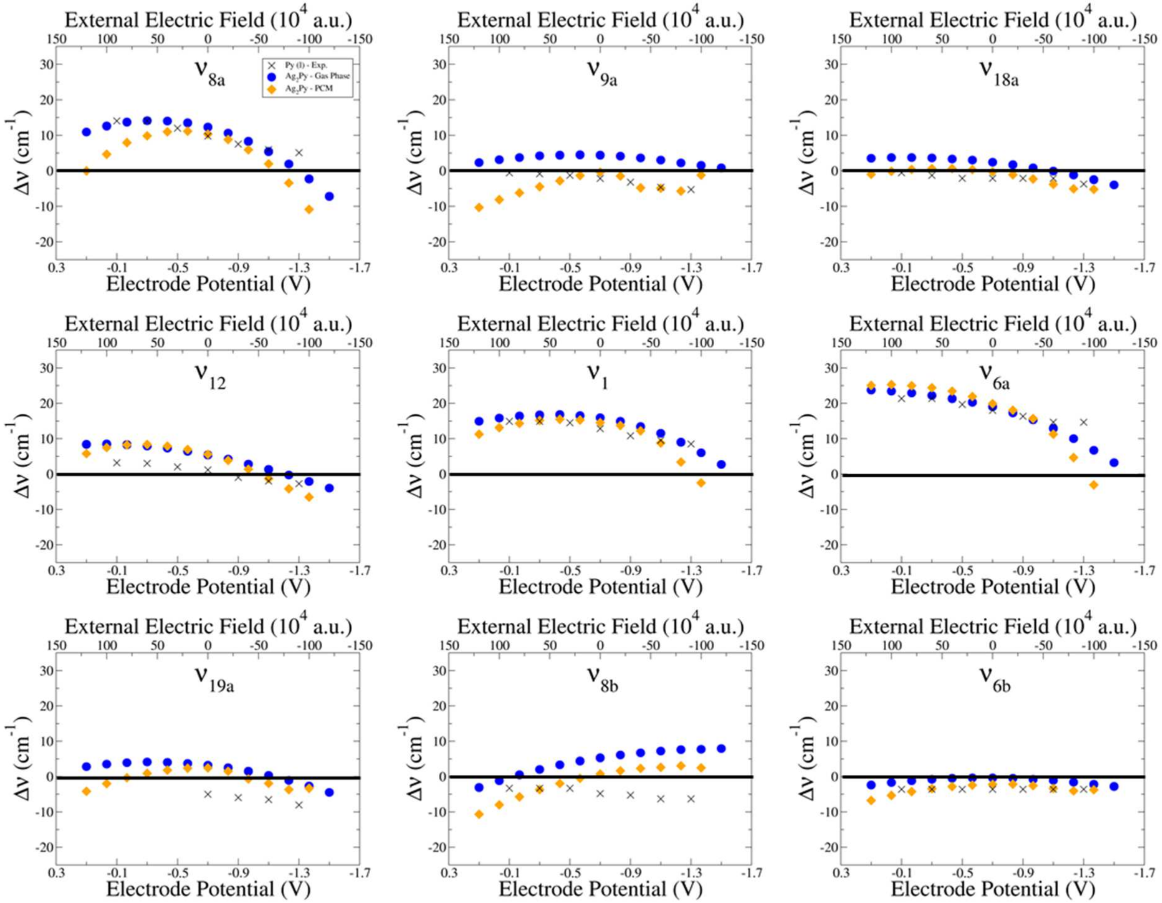

Supplementary Figure S26 Experimental wavenumber shifts $\Delta\upsilon$ (crosses) and B3LYP/LanL2DZ/6-311G(d,p) calculated values under an external electric field from isolated (blue) and solvated (PCM, orange) $[Ag_2Py]^0$ complex.

$[Ag_2Py]^0$ PW91/LanL2DZ/6-311G(d,p)

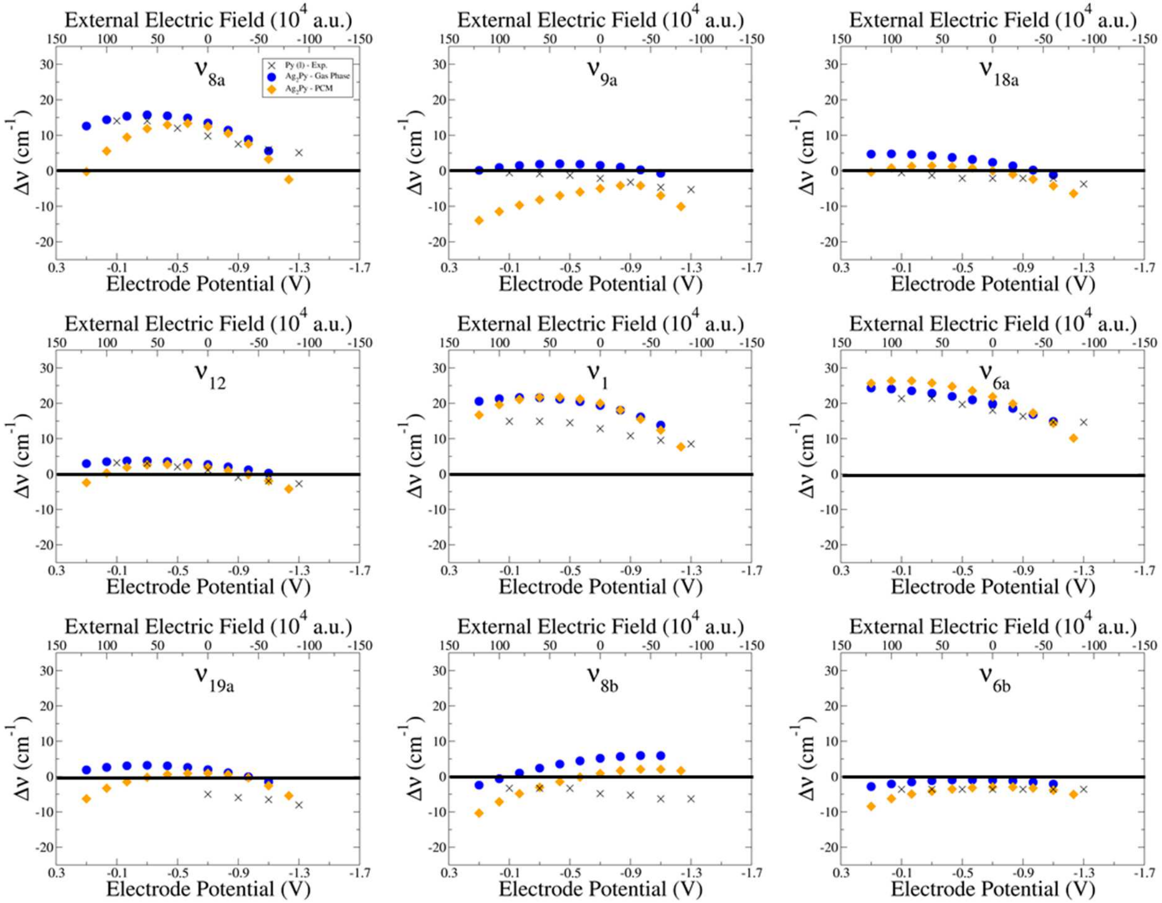

Supplementary Figure S27 Experimental wavenumber shifts $\Delta\upsilon$ (crosses) and PW91/LanL2DZ/6-311G(d,p) calculated values under an external electric field from isolated (blue) and solvated (PCM, orange) $[Ag_2Py]^0$ complex.

$[Ag_2Py]^0$ M06-HF/LanL2DZ/6-311G(d,p)

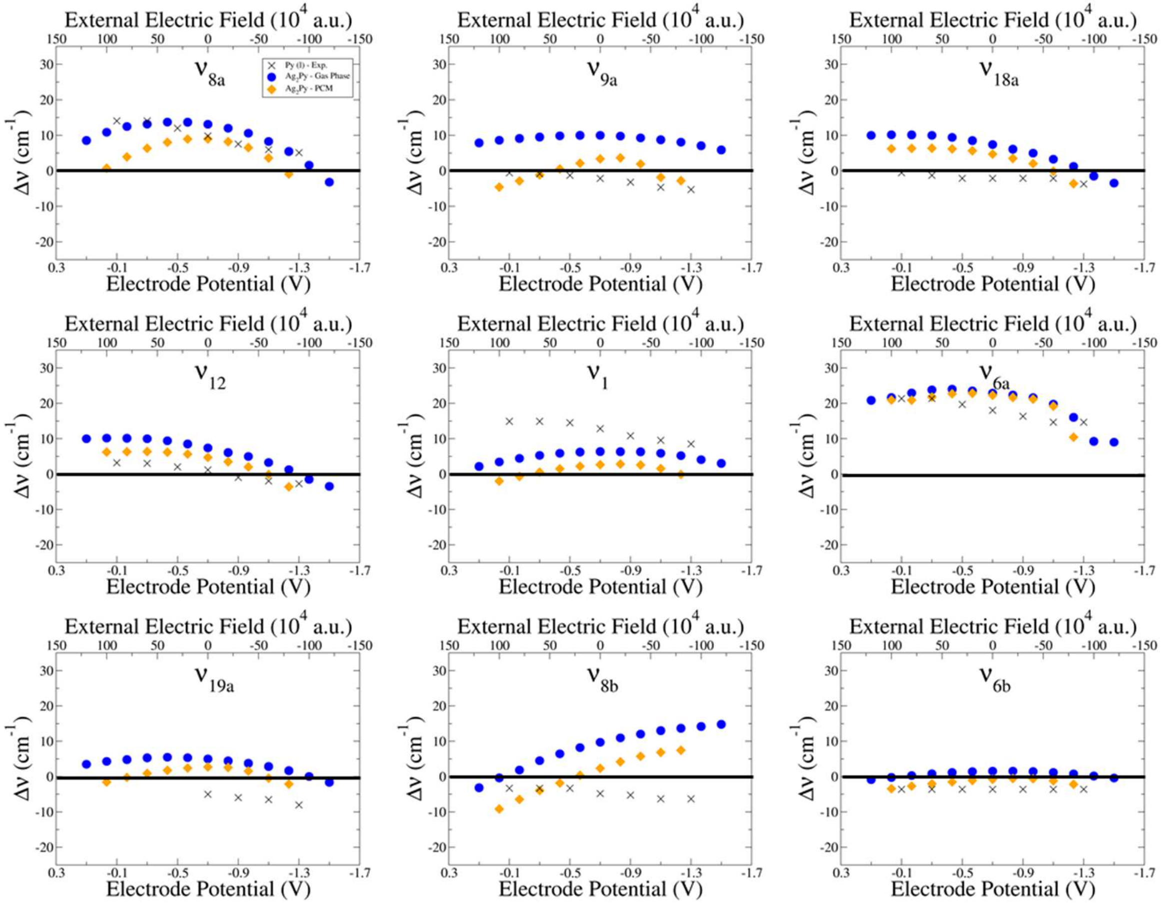

Supplementary Figure S28 Experimental wavenumber shifts $\Delta\upsilon$ (crosses) and M06-HF/LanL2DZ/6-311G(d,p) calculated values under an external electric field from isolated (blue) and solvated (PCM, orange) $[Ag_2Py]^0$ complex.
