# Supplementary figures and images for: Theoretical Approaches for Modeling the Effect of the Electrode Potential in the SERS Vibrational Wavenumbers of Pyridine Adsorbed on a Charged Silver Surface
Source: Front Chem. 2019 Jun 5;7:423. doi: 10.3389/fchem.2019.00423 (PMC6560080; doi:10.3389/fchem.2019.00423)

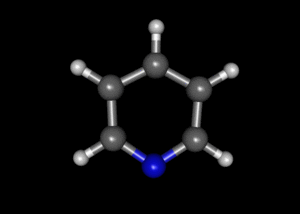

Supplement: Supplementary file 2 [file Data_Sheet_2.ZIP › 1.gif]

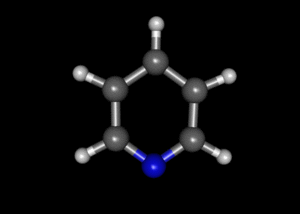

Supplement: Supplementary file 2 [file Data_Sheet_2.ZIP › 6a.gif]

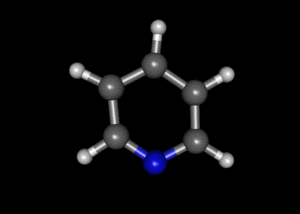

Supplement: Supplementary file 2 [file Data_Sheet_2.ZIP › 6b.gif]

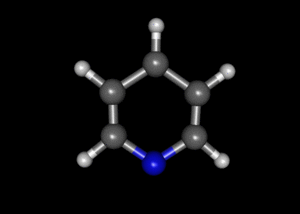

Supplement: Supplementary file 2 [file Data_Sheet_2.ZIP › 8a.gif]

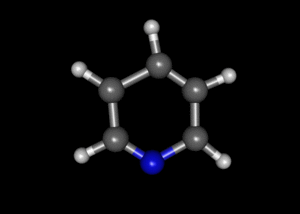

Supplement: Supplementary file 2 [file Data_Sheet_2.ZIP › 8b.gif]

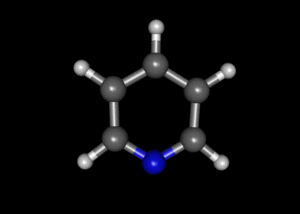

Supplement: Supplementary file 2 [file Data_Sheet_2.ZIP › 9a.gif]

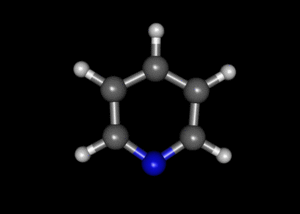

Supplement: Supplementary file 2 [file Data_Sheet_2.ZIP › 12.gif]

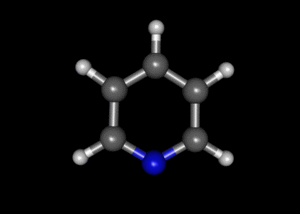

Supplement: Supplementary file 2 [file Data_Sheet_2.ZIP › 18a.gif]

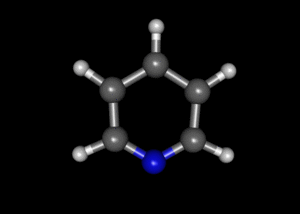

Supplement: Supplementary file 2 [file Data_Sheet_2.ZIP › 19a.gif]
